# Polymorphisms within Autophagy-Related Genes as Susceptibility Biomarkers for Multiple Myeloma: A Meta-Analysis of Three Large Cohorts and Functional Characterization

**DOI:** 10.3390/ijms24108500

**Published:** 2023-05-09

**Authors:** Esther Clavero, José Manuel Sanchez-Maldonado, Angelica Macauda, Rob Ter Horst, Belém Sampaio-Marques, Artur Jurczyszyn, Alyssa Clay-Gilmour, Angelika Stein, Michelle A. T. Hildebrandt, Niels Weinhold, Gabriele Buda, Ramón García-Sanz, Waldemar Tomczak, Ulla Vogel, Andrés Jerez, Daria Zawirska, Marzena Wątek, Jonathan N. Hofmann, Stefano Landi, John J. Spinelli, Aleksandra Butrym, Abhishek Kumar, Joaquín Martínez-López, Sara Galimberti, María Eugenia Sarasquete, Edyta Subocz, Elzbieta Iskierka-Jażdżewska, Graham G. Giles, Malwina Rybicka-Ramos, Marcin Kruszewski, Niels Abildgaard, Francisco García Verdejo, Pedro Sánchez Rovira, Miguel Inacio da Silva Filho, Katalin Kadar, Małgorzata Razny, Wendy Cozen, Matteo Pelosini, Manuel Jurado, Parveen Bhatti, Marek Dudzinski, Agnieszka Druzd-Sitek, Enrico Orciuolo, Yang Li, Aaron D. Norman, Jan Maciej Zaucha, Rui Manuel Reis, Miroslaw Markiewicz, Juan José Rodríguez Sevilla, Vibeke Andersen, Krzysztof Jamroziak, Kari Hemminki, Sonja I. Berndt, Vicent Rajkumar, Grzegorz Mazur, Shaji K. Kumar, Paula Ludovico, Arnon Nagler, Stephen J. Chanock, Charles Dumontet, Mitchell J. Machiela, Judit Varkonyi, Nicola J. Camp, Elad Ziv, Annette Juul Vangsted, Elizabeth E. Brown, Daniele Campa, Celine M. Vachon, Mihai G. Netea, Federico Canzian, Asta Försti, Juan Sainz

**Affiliations:** 1 Hematology Department, Virgen de las Nieves University Hospital, 18012 Granada, Spain; eclaverosa@hotmail.com (E.C.); manuel.jurado.sspa@juntadeandalucia.es (M.J.); 2 Genomic Oncology Area, GENYO, Centre for Genomics and Oncological Research, Pfizer/University of Granada/Andalusian Regional Government, PTS, 18016 Granada, Spain; josemanuel.sanchez@genyo.es; 3 Instituto de Investigación Biosanataria IBs, Granada, 18014 Granada, Spain; 4 Genomic Epidemiology Group, German Cancer Research Center (DKFZ), 69120 Heidelberg, Germany; angelicamacauda@gmail.com (A.M.); a.stein@dkfz.de (A.S.); f.canzian@dkfz.de (F.C.); 5 Department of Internal Medicine and Radboud Center for Infectious Diseases, Radboud University Medical Center, 6525 GA Nijmegen, The Netherlands; rob.terhorst@radboudumc.nl (R.T.H.); yang.li@helmholtz-hzi.de (Y.L.); mihai.netea@radboudumc.nl (M.G.N.); 6 CeMM Research Center for Molecular Medicine of the Austrian Academy of Sciences, 1090 Vienna, Austria; 7 Life and Health Sciences Research Institute (ICVS), School of Medicine, University of Minho, 4710-057 Braga, Portugal; mbmarques@med.uminho.pt (B.S.-M.); pludovico@med.uminho.pt (P.L.); 8 Plasma Cell Dyscrasias Center, Department of Hematology, Jagiellonian University Medical College, 31-066 Kraków, Poland; mmjurczy@cyf-kr.edu.pl; 9 Department of Biostatistics and Epidemiology, Arnold School of Public Health, University of South Carolina, Greenville, SC 29208, USA; claygila@mailbox.sc.edu; 10 Division of Epidemiology, Department of Health Sciences Research, Mayo Clinic, Rochester, MN 55902, USA; aarondeannorman@gmail.com (A.D.N.); vachon.celine@mayo.edu (C.M.V.); 11 Department of Lymphoma–Myeloma, Division of Cancer Medicine, The University of Texas MD Anderson Cancer Center, Houston, TX 77030, USA; mhildebr@mdanderson.org; 12 Myeloma Institute, University of Arkansas for Medical Sciences, Little Rock, AR 72205, USA; niels.weinhold@med.uni-heidelberg.de; 13 Department of Internal Medicine V, University of Heidelberg, 69120 Heidelberg, Germany; 14 Haematology Unit, Department of Clinical and Experimental Medicine, University of Pisa/AOUP, 56126 Pisa, Italy; ga.buda@libero.it (G.B.); sara.galimberti@med.unipi.it (S.G.); e.orciuolo@ao-pisa.toscana.it (E.O.); 15 Diagnostic Laboratory Unit in Hematology, University Hospital of Salamanca, IBSAL, CIBERONC, Centro de Investigación del Cáncer-IBMCC (USAL-CSIC), 37007 Salamanca, Spain; rgarcia@usal.es (R.G.-S.); a9136@usal.es (M.E.S.); 16 Department of Hematooncology and Bone Marrow Transplantation, Medical University of Lublin, 20-059 Lublin, Poland; waldemar.tomczak@umlub.pl; 17 National Research Centre for the Working Environment, DK-2100 Copenhagen, Denmark; ubv@nfa.dk; 18 Department of Hematology, Experimental Hematology Unit, Vall d’Hebron Institute of Oncology (VHIO), University Hospital Vall d’Hebron, 08035 Barcelona, Spain; anjecayu@gmail.com; 19 Department of Hematology, University Hospital, 30-688 Kraków, Poland; dariafm@poczta.fm; 20 Holycross Medical Oncology Center, 25-735 Kielce, Poland; marzena.watek@wp.pl; 21 Institute of Hematology and Transfusion Medicine, 00-791 Warsaw, Poland; 22 Division of Cancer Epidemiology and Genetics, National Cancer Institute, National Institutes of Health, Bethesda, MD 20892, USA; hofmannjn@mail.nih.gov (J.N.H.); berndts@mail.nih.gov (S.I.B.); chanocks@mail.nih.gov (S.J.C.); machielamj@mail.nih.gov (M.J.M.); 23 Department of Biology, University of Pisa, 56126 Pisa, Italy; stefano.landi@unipi.it (S.L.); daniele.campa@unipi.it (D.C.); 24 Division of Population Oncology, BC Cancer, Vancouver, BC V5Z 4E6, Canada; jspinelli@bccrc.ca; 25 School of Population and Public Health, University of British Columbia, Vancouver, BC V6T 1Z4, Canada; 26 Department of Cancer Prevention and Therapy, Wroclaw Medical University, 50-367 Wroclaw, Poland; aleksandra.butrym@gmail.com; 27 Alfred Sokolowski Specialist Hospital in Walbrzych Oncology Support Centre for Clinical Trials, 58-309 Walbrzych, Poland; 28 Institute of Bioinformatics, International Technology Park, Bangalore 560066, India; abhishek@ibioinformatics.org; 29 Manipal Academy of Higher Education (MAHE), Manipal 576104, India; 30 Hospital 12 de Octubre, Complutense University, CNIO, CIBERONC, 28041 Madrid, Spain; jmarti01@ucm.es; 31 Department of Hematology, Military Institute of Medicine, 04-141 Warsaw, Poland; suboczka@poczta.onet.pl; 32 Department of Hematology, Medical University of Lodz, 90-419 Lodz, Poland; elzbieta.iskierka-jazdzewska@umed.lodz.pl; 33 Cancer Epidemiology Division, Cancer Council Victoria, Melbourne, VIC 3004, Australia; graham.giles@cancervic.org.au; 34 Centre for Epidemiology and Biostatistics, School of Population and Global Health, The University of Melbourne, Melbourne, VIC 3010, Australia; 35 Precision Medicine, School of Clinical Sciences at Monash Health, Monash University, Clayton, VIC 3168, Australia; 36 Department of Hematology, Specialist Hospital No. 1 in Bytom, Academy of Silesia, Faculty of Medicine, 40-055 Katowice, Poland; malwina.rybicka@gmail.com; 37 Department of Hematology, University Hospital No. 2, 85-168 Bydgoszcz, Poland; marcin.kruszewski5@wp.pl; 38 Department of Hematology, Odense University Hospital, DK-5000 Odense, Denmark; niels.abildgaard@rsyd.dk; 39 Department of Medical Oncology, Complejo Hospitalario de Jaén, 23007 Jaén, Spain; francisco.garcia.verdejo.sspa@juntadeandalucia.es (F.G.V.); oncopsr@yahoo.es (P.S.R.); 40 Division of Molecular Genetic Epidemiology, German Cancer Research Center (DKFZ), Im Neuenheimer Feld 580, D-69120 Heidelberg, Germany; m.dasilvafilho@dkfz-heidelberg.de; 41 St Johns Hospital, 62769 Budapest, Hungary; kadarkataeszter@gmail.com; 42 Department of Hematology, Rydygier Hospital, 31-826 Cracow, Poland; m.razny@wp.pl; 43 Division of Hematology/Oncology, Department of Medicine, School of Medicine, Department of Pathology, School of Medicine, Susan and Henry Samueli College of Health Sciences, Chao Family Comprehensive Cancer Center, University of California at Irvine, Irvine, CA 92697, USA; wcozen@hs.uci.edu; 44 U.O. Dipartimento di Ematologia, Azienda USL Toscana Nord Ovest, 57124 Livorno, Italy; matteo.pelosini@ao-pisa.toscana.it; 45 Department of Medicine, University of Granada, 18012 Granada, Spain; 46 Cancer Control Research, BC Cancer, Vancouver, BC V5Z 4E6, Canada; pbhatti@bccrc.ca; 47 Program in Epidemiology, Public Health Sciences Division, Fred Hutchinson Cancer Research Center, Seattle, WA 98109, USA; 48 Department of Hematology, Institute of Medical Sciences, College of Medical Sciences, University of Rzeszow, 35-310 Rzeszow, Poland; marekdudzi@gmail.com (M.D.); mir.markiewicz@wp.pl (M.M.); 49 Department of Lymphoproliferative Diseases, Maria Skłodowska Curie National Research Institute of Oncology, 02-781 Warsaw, Poland; adruzd@coi.waw.pl; 50 Centre for Individualised Infection Medicine (CiiM) & TWINCORE, Joint Ventures between the Helmholtz-Centre for Infection Research (HZI) and the Hannover Medical School (MHH), 30625 Hannover, Germany; 51 Genetic Epidemiology and Risk Assessment Program, Mayo Clinic Comprehensive Cancer Center, Division of Biomedical Statistics and Informatics, Department of Health Sciences Research, Mayo Clinic, Rochester, MN 55902, USA; 52 Department of Hematology and Transplantology, Medical University of Gdansk, 80-210 Gdansk, Poland; jzaucha@gumed.edu.pl; 53 Life and Health Sciences Research Institute (ICVS), School of Health Sciences, University of Minho, 4710-057 Braga, Portugal and ICVS/3B’s-PT Government Associate Laboratory, 4710-057 Braga/Guimarães, Portugal; rreis@med.uminho.pt; 54 Molecular Oncology Research Center, Barretos Cancer Hospital, Barretos 14784-400, Brazil; 55 Department of Hematology, Hospital del Mar, 08003 Barcelona, Spain; jrodsevilla@gmail.com; 56 Molecular Diagnostics and Clinical Research Unit, Institute of Regional Health Research, University Hospital of Southern Denmark, DK-6200 Aabenraa, Denmark; vibeke.andersen1@rsyd.dk; 57 Department of Hematology, Transplantology and Internal Medicine, Medical University of Warsaw, 02-097 Warsaw, Poland; krzysztof.jamroziak@wp.pl; 58 Division of Cancer Epidemiology, German Cancer Research Center (DKFZ), Im Neuenheimer Feld 280, 69120 Heidelberg, Germany; k.hemminki@dkfz-heidelberg.de; 59 Faculty of Medicine and Biomedical Center in Pilsen, Charles University in Prague, 30605 Pilsen, Czech Republic; 60 Division of Hematology, Department of Internal Medicine, Mayo Clinic, Rochester, MN 55902, USA; rajkumar.vincent@mayo.edu (V.R.); kumar.shaji@mayo.edu (S.K.K.); 61 Department of Internal Diseases, Occupational Medicine, Hypertension and Clinical Oncology, Wroclaw Medical University, 50-368 Wroclaw, Poland; grzegorzmaz@yahoo.com; 62 Hematology Division, Chaim Sheba Medical Center, Tel Hashomer 52621, Israel; a.nagler@sheba.health.gov.il; 63 UMR INSERM 1052/CNRS 5286, University of Lyon, Hospices Civils de Lyon, 69008 Lyon, France; charles.dumontet@chu-lyon.fr; 64 Semmelweis University, 1083 Budapest, Hungary; varkonyi.judit@med.semmelweis-univ.hu; 65 Division of Hematology, Huntsman Cancer Institute, University of Utah, Salt Lake City, UT 84112, USA; nicki.camp@hci.utah.edu; 66 Department of Medicine, University of California San Francisco Helen Diller Family Comprehensive Cancer Center, San Francisco, CA 94143, USA; elad.ziv@ucsf.edu; 67 Department of Hematology, Rigshospitalet, Copenhagen University, DK-2100 Copenhagen, Denmark; annette.juul.vangsted@regionh.dk; 68 Department of Pathology, Heersink School of Medicine, The University of Alabama at Birmingham, Birmingham, AL 35294, USA; elizabethbrown@uabmc.edu; 69 Department for Immunology & Metabolism, Life and Medical Sciences Institute (LIMES), University of Bonn, 53115 Bonn, Germany; 70 Division of Pediatric Neurooncology, German Cancer Research Center (DKFZ), German Cancer Consortium (DKTK), 69120 Heidelberg, Germany; a.foersti@kitz-heidelberg.de; 71 Hopp Children’s Cancer Center (KiTZ), 69120 Heidelberg, Germany; 72 Department of Biochemistry and Molecular Biology I, University of Granada, 18071 Granada, Spain

**Keywords:** multiple myeloma, autophagy, genetic variants, genetic susceptibility

## Abstract

**Simple Summary:**

We investigated the influence of autophagy-related variants in modulating Multiple Myeloma (MM) risk through a meta-analysis of germline genetic data on 234 autophagy-related genes from three independent study populations including 13,387 subjects of European ancestry (6863 MM patients and 6524 controls) and examined the functional mechanisms behind the observed associations. We identified SNPs within the six *CD46*, *IKBKE*, *PARK2*, *ULK4*, *ATG5*, and *CDKN2A* loci associated with MM risk and observed that their effect on disease risk was mediated by specific subsets of immune cells, as well as vitamin D3-, MCP-2-, and IL20-dependent mechanisms.

**Abstract:**

Multiple myeloma (MM) arises following malignant proliferation of plasma cells in the bone marrow, that secrete high amounts of specific monoclonal immunoglobulins or light chains, resulting in the massive production of unfolded or misfolded proteins. Autophagy can have a dual role in tumorigenesis, by eliminating these abnormal proteins to avoid cancer development, but also ensuring MM cell survival and promoting resistance to treatments. To date no studies have determined the impact of genetic variation in autophagy-related genes on MM risk. We performed meta-analysis of germline genetic data on 234 autophagy-related genes from three independent study populations including 13,387 subjects of European ancestry (6863 MM patients and 6524 controls) and examined correlations of statistically significant single nucleotide polymorphisms (SNPs; *p* < 1 × 10^−9^) with immune responses in whole blood, peripheral blood mononuclear cells (PBMCs), and monocyte-derived macrophages (MDM) from a large population of healthy donors from the Human Functional Genomic Project (HFGP). We identified SNPs in six loci, *CD46*, *IKBKE*, *PARK2*, *ULK4*, *ATG5*, and *CDKN2A* associated with MM risk (*p* = 4.47 × 10^−4^−5.79 × 10^−14^). Mechanistically, we found that the *ULK4*_rs6599175_ SNP correlated with circulating concentrations of vitamin D3 (*p* = 4.0 × 10^−4^), whereas the *IKBKE*_rs17433804_ SNP correlated with the number of transitional CD24^+^CD38^+^ B cells (*p* = 4.8 × 10^−4^) and circulating serum concentrations of Monocyte Chemoattractant Protein (MCP)-2 (*p* = 3.6 × 10^−4^). We also found that the *CD46*_rs1142469_ SNP correlated with numbers of CD19^+^ B cells, CD19^+^CD3^−^ B cells, CD5^+^IgD^−^ cells, IgM^−^ cells, IgD^−^IgM^−^ cells, and CD4^−^CD8^−^ PBMCs (*p* = 4.9 × 10^−4^−8.6 × 10^−4^) and circulating concentrations of interleukin (IL)-20 (*p* = 0.00082). Finally, we observed that the *CDKN2A*_rs2811710_ SNP correlated with levels of CD4^+^EMCD45RO^+^CD27^−^ cells (*p* = 9.3 × 10^−4^). These results suggest that genetic variants within these six loci influence MM risk through the modulation of specific subsets of immune cells, as well as vitamin D3^−^, MCP-2^−^, and IL20-dependent pathways.

## 1. Introduction

Multiple myeloma (MM) is a relatively common and incurable hematological malignancy arising from post–germinal mature B cells and it is characterized by the presence of proliferating plasma cells in the bone marrow that secrete specific monoclonal immunoglobulin (also called M-protein) [1]. Given the exacerbated production of immunoglobulin, MM patients often suffer from a concomitant decrease in normal immunoglobulins [2] that causes immune dysfunction, increases susceptibility to opportunistic infections, and impacts on disease severity and prognosis [3,4].

Although the high production of monoclonal immunoglobulins occurring in MM invariably results in the presence of a high amount of unfolded or misfolded proteins that might be toxic for MM cells in the bone marrow, recent studies demonstrated that plasma cells are able to exploit molecular pathways to protect themselves from damage caused by toxic proteins [5]. These molecular pathways include the activation of unfolded protein response (UPR) [6,7,8], heat protein chaperones [9], aggresome formation [10], and the induction of cellular autophagy [5]. Autophagy is a lysosome-dependent catabolic degradation process by which cells remove toxic aggregated cytosolic proteins and malfunctioning organelles. It has been well-documented that autophagy not only is an autonomous mechanism that modulates cell homeostasis at basal level, but it is also involved in B-cell development and proliferation [11], cell survival [12,13], apoptosis [14,15], tumorigenesis, anti-tumoral immune responses [16], and resistance to chemotherapeutic agents. In this regard, a growing number of studies have suggested that autophagy shapes anti-tumoral immune responses by acting at multiple levels [17]. This includes the promotion of signals to activate phagocytosis of tumor cells [18,19], as well as signals to induce myeloid cell recruitment [20], MHC-class-I and -II presentation [21], B- and T-cell activation, development, maintenance [11,22], and self-tolerance [23]. Strikingly, experimental studies have demonstrated that the inhibition of autophagy enhances the sensitivity of MM cells to a number of anticancer agents and induces MM cell death [24,25]. Based on these findings, it has been suggested that autophagy could be targeted to treat MM [5,24,26] and recent studies and clinical trials have indeed confirmed the therapeutic implications of new and less toxic autophagy inhibitors in MM, used in combination with current anti-MM drugs [27,28,29].

In support of a possible role of autophagy in MM, it was reported that aberrant expression of autophagy-related genes is associated with cancer development [30] and that multiple activators of autophagy or specific autophagy-related genes are commonly found in cancer-associated regions [31]. Interestingly, it was also described that expression of autophagy-related genes influences the response to conventional treatments in MM [32,33,34] and, thereby, disease progression [35,36], arguing that they may be useful to predict disease risk [37]. However, despite these findings suggesting a key role of autophagy in the etiology of MM and the existence of a genetic component controlling this catalytic process in MM, so far only *ULK4*, *ATG5,* and *CDKN2A* polymorphisms have been suggested to have an impact on the risk of MM [38,39]. Therefore, it is vital to perform a systematic analysis of autophagy-related markers to identify new susceptibility variants for MM and validate those associations already reported.

In this context, the aim of this study was to comprehensively evaluate the impact of common genetic variation of 234 autophagy-related genes in determining the risk of developing MM. We also assessed the influence of the most promising markers on modulating immune responses in whole blood, peripheral blood mononuclear cells (PBMCs), and monocyte-derived macrophages (MDM) from a large population of healthy donors from the Human Functional Genomic Project (HFGP). Additionally, we measured the autophagy flux in an independent population.

## 2. Results

### 2.1. Association of Autophagy-Related Polymorphisms with the Risk of Developing MM

This study included 8719 individuals (3916 MM cases and 4803 controls) from the German GWAS consisting of 1512 MM patients and 2107 controls and the InterLymph MM GWAS that included 2404 MM cases and 2696 healthy controls. Selected polymorphisms showed no deviation from HWE (*p* < 0.001), either in the German GWAS or in the InterLymph MM GWAS. The association analysis of the German cohort showed that 440 independent SNPs (r^2^ < 0.1) were significantly associated with MM risk at *p* ≤ 0.05. The association of these SNPs with MM risk was then validated through meta-analysis with data from the InterLymph MM GWAS. The meta-analysis of these large independent studies confirmed the association of 12 genetic variants within the *ATG5*, *CD46*, *CDKN2A*, *CTSD*, *HSPB8*, *IKBKE*, *PARK2*, *RPTOR*, *ULK4*, and *USP10* loci with MM risk (Table 1), with loci in *ULK4* and *ATG5* reaching the highest level of statistical significance.

Although the lack of significant heterogeneity between both study populations suggested that the association found for these 12 SNPs might represent true associations, we decided to replicate these findings in a third independent population ascertained through the IMMEnSE consortium that included 2696 MM cases and 1701 controls. Results are reported in Table 2. After correction for multiple testing (*p*_Bonferroni_ = 1.14 × 10^−4^), we could confirm the previously reported association for *ULK4*, *ATG5*, and *CDKN2A* polymorphisms. Importantly, we also found that the association of the *IKBKE*_rs17433804_ SNP with the risk of MM was observed after multiple testing correction, which suggests that this gene could represent a new susceptibility locus for MM. In addition, although the associations were borderline significant after correction for multiple comparisons, we found of interest the associations of *CD46* and *PARK2* variants with the risk of developing MM. Although these associations need to be further validated, it was important to confirm that, with the exception of the *CDKN2A*, all the association signals included several SNPs (LD blocks), which reinforced the role of these loci in determining disease risk (Supplementary Figure S1A–F).

### 2.2. Functional Relevance of Autophagy-Related SNPs

Given the relatively strong association of the genetic variants within the *CD46*, *IKBKE*, *PARK2*, *ULK4*, *ATG5*, and *CDKN2A* loci on MM risk, we explored whether these variants could modulate host immune responses, serum steroid hormones, circulating immunological proteins, and blood-derived cell populations using data from the HFGP population. Although the genetic association of the *USP10*_rs7202154_ SNP with MM risk was modest, we decided to include it in the functional analysis due to the known role of USP proteins on the modulation of MM cell apoptosis.

**Figure 1:**
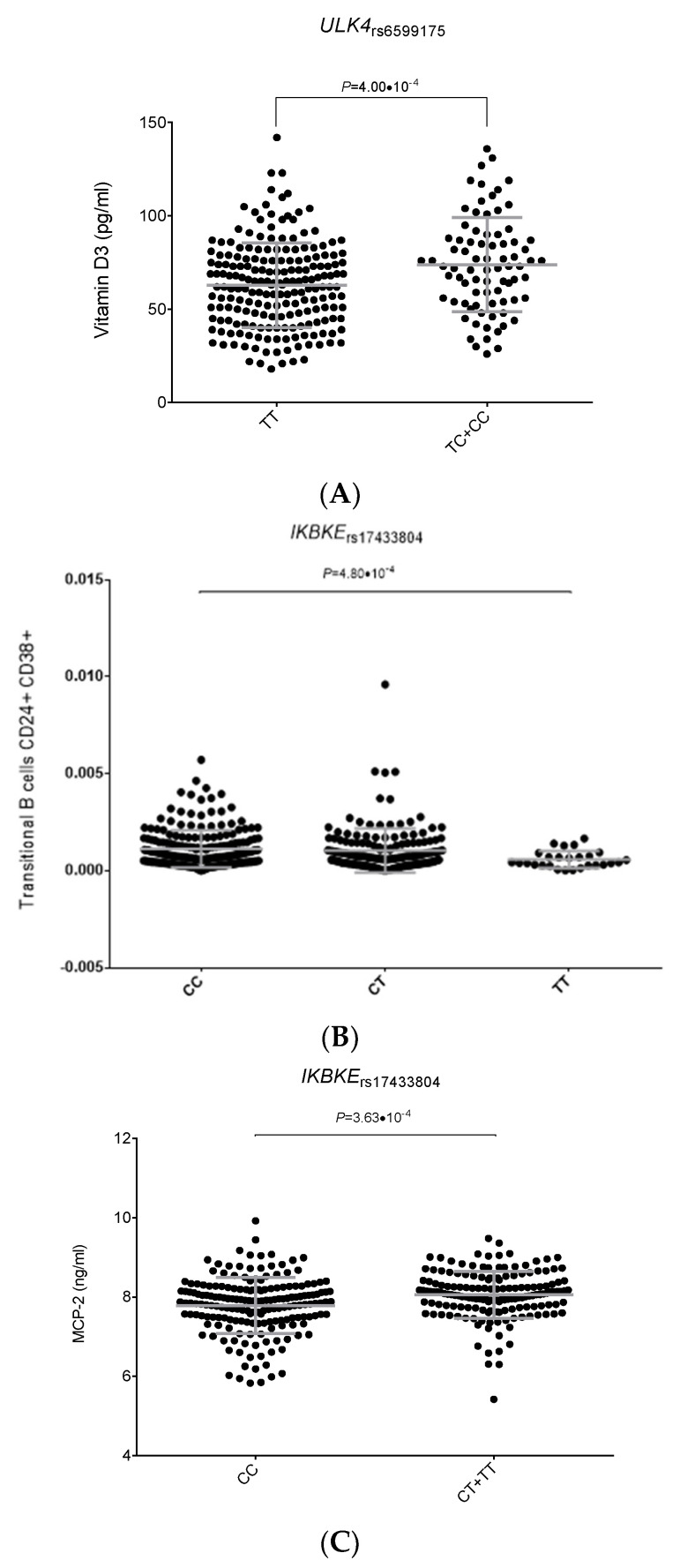
Functional impact of the *ULK4*_rs6599175_ and *IKBKE*_rs17433804_ SNPs [A-C]. (**A**) Vitamin D3 levels (pg/mL) according to the *ULK4*_rs6599175_ SNP; (**B**) Numbers of transitional CD24^+^CD38^+^ B cells according to the *IKBKE*_rs17433804_ SNP; (**C**) Serum levels of MCP-2 according to the *IKBKE*_rs17433804_ SNP.

Interestingly, we found a significant association between the *ULK4*_rs6599175_ SNP and circulating concentrations of vitamin D3 (*p* = 4.0 × 10^−4^, Figure 1A), which suggests a possible involvement of this SNP in modulating MM risk through a vitamin D-dependent mechanism. We also observed that the presence of the *IKBKE*_rs17433804C_ allele was associated with increased numbers of transitional CD24^+^CD38^+^ B cells (*p* = 4.8 × 10^−4^, Figure 1B), whereas carriers of the *IKBKE*_rs17433804C/C_ genotype showed decreased circulating serum concentrations of MCP-2 (*p* = 3.6 × 10^−4^; Figure 1C), a chemotactic molecule involved in the activation of multiple immune cells and linked to MM cell migration.

**Figure 2:**
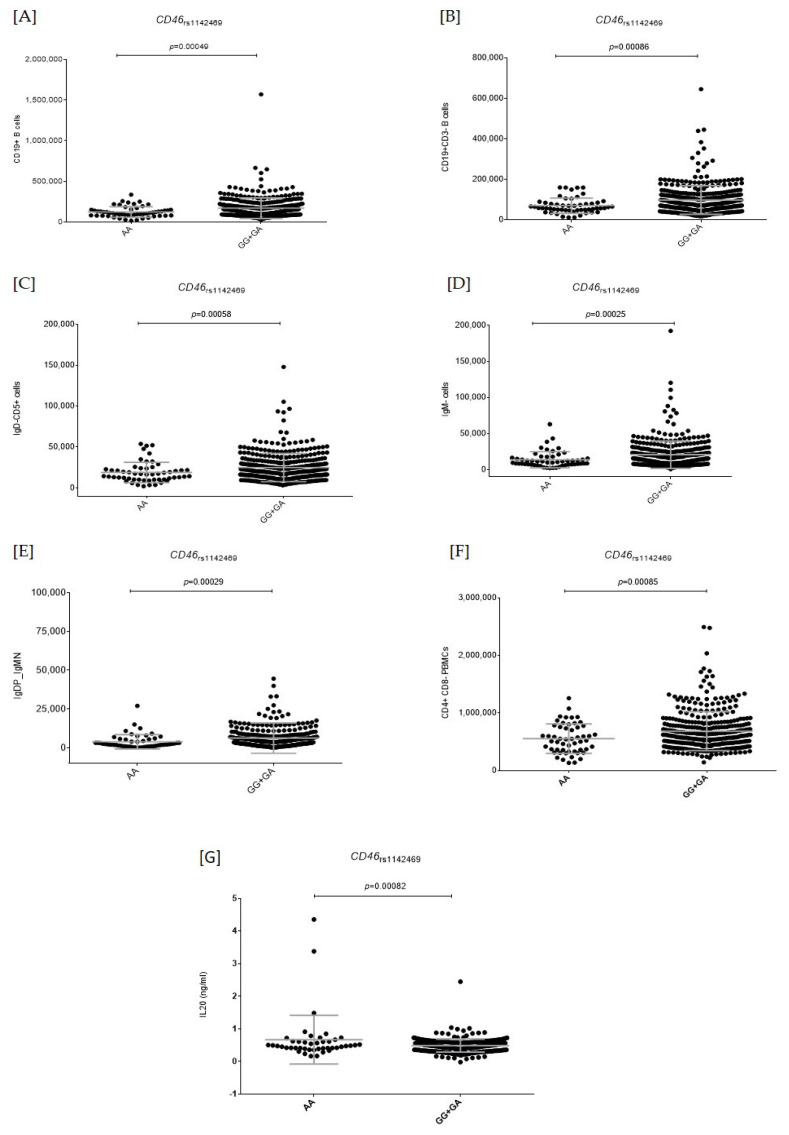
Functional impact of the *CD46*_rs1142469_ SNP (**A**–**G**). (**A**) Numbers of CD19^+^ B cells according to the *CD46*_rs1142469_ SNP; (**B**) Numbers of CD19^+^CD3^−^ B cells according to the *CD46*_rs1142469_ SNP; (**C**) Numbers of IgD^−^CD5^+^ cells according to the *CD46*_rs1142469_ SNP; (**D**) Numbers of IgM^−^ cells according to the *CD46*_rs1142469_ SNP; (**E**) Numbers of IgD^+^IgM^−^ cells according to the *CD46*_rs1142469_ SNP; (**F**) Numbers of CD4^+^CD8^−^ PBMCs according to the *CD46*_rs1142469_ SNP; and (**G**) Serum IL20 levels (ng/mL) according to the *CD46*_rs1142469_ SNP.

We also found that homozygous carriers of the *CD46*_rs1142469A_ allele, which was associated with an increased risk of MM, had decreased numbers of CD19^+^ B cells, CD19^+^CD3^−^ B cells, CD5^+^IgD^−^ cells, IgM^−^ cells, IgD^−^IgM^−^ cells, and CD4^−^CD8^−^ PBMCs compared with those subjects carrying the G allele (*p* = 0.00025–0.00086; Figure 2A–F).

Moreover, we found that homozygous carriers of the *CD46*_rs1142469A_ risk allele had increased circulating concentrations of IL20 compared with those subjects carrying the G allele (*p* = 0.00082; Figure 2G), which suggests a role of this angiogenic cytokine in modulating MM risk.

**Figure 3:**
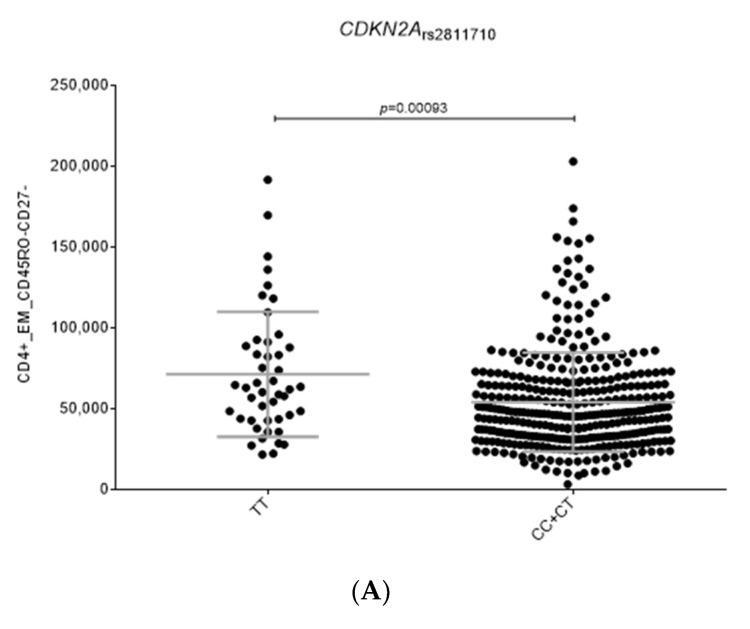

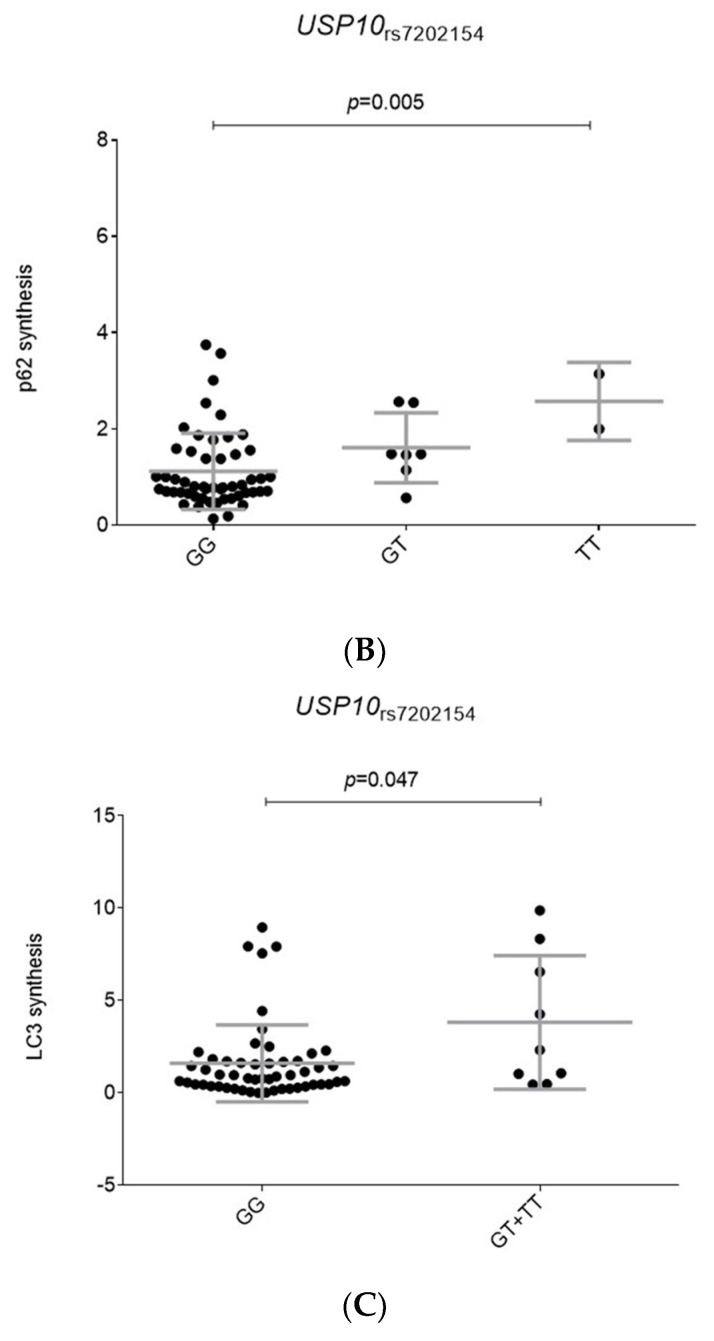
Functional impact of the *CDKN2A*_rs2811710_ and *USP10*_rs7202154_ SNPs. (**A**) Numbers of CD4^+^ Effector Memory CD45RO^−^CD27^−^ cells according to the *CDKN2A*_rs2811710_ SNP; (**B**) p62 synthesis according to the *USP10*_rs7202154_ SNP; (**C**) LC3 synthesis according to the *USP10*_rs7202154_ SNP.

In addition, we observed that carriers of the *CDKN2A*_rs2811710C_ allele had decreased levels of CD4^+^EMCD45RO^+^CD27^−^ cells (*p* = 0.00093; Figure 3A), which constitutes a subset of memory T cells that does not require co-stimulation for T-cell receptors to display a high antigen recall response. Finally, we found that carriers of the *USP10*_rs7202154G/G_ genotype had decreased synthesis of p62 and LC3-II compared to non-carriers (*p* = 0.005 and *p* = 0.047; Figure 3B,C). Given that p62 serves as a useful marker for the induction of autophagy, clearance of protein aggregates, and the inhibition of autophagy and LC3-II serves to track the binding of p62 and subsequent recruitment of autophagosomes, this result might suggest that this genetic variant could be involved in determining autophagy activation. Given that the association of the *USP10* SNP with autophagy flux markers (LC3-II/Actin ratio) did not remain statistically significant after correction for multiple testing, additional studies are needed to confirm whether the weak association of this SNP with MM risk might be mediated by the regulation of the autophagy flux. No functional effect on host immune responses or autophagy flux was detected for the remaining selected SNPs, which suggests that the effect of these variants on MM risk is not mediated by these biological processes.

## 3. Discussion

To the best of our knowledge this is the first population-based case-control study comprehensively assessing the role of autophagy-related SNPs in modulating the risk of developing MM. After a comprehensive meta-analysis of three large independent studies, we found three novel significant associations between MM risk and SNPs within *CD46*, *IKBKE*, and *PARK2*, in addition to confirming the known associations with MM-susceptibility genes *ULK4*, *ATG5*, and *CDKN2A* loci.

As the most interesting finding, we found an association of the *CD46*_rs1142469_ SNP with the risk of developing MM. The *CD46* locus is located on chromosome 1q32 and has been involved in pathogen recognition and in the differentiation of CD4^+^ cells into T-regulatory 1 cells that suppress immune responses by secreting IL10. The *CD46*_rs1142469_ SNP is an intronic variant that acts as eQTL for the *CD46* in multiple tissues [40,41] and lymphoblastoid cell lines [41] and it is in LD with three neighboring SNPs that confirm the association signal of the *CD46* locus with MM risk. Our functional results also showed that homozygous carriers of the *CD46*_rs1142469A_ risk allele had decreased numbers of CD19^+^ B cells, CD19^+^CD3^−^ B cells, CD5^+^IgD^−^, IgM^−^ cells, IgD^−^, IgM^−^ cells, and CD4^−^, CD8^−^ PBMCs, but also increased circulating concentrations of IL20. This latter observation is consistent with previous studies, which reported that IL20 is highly expressed in MM patients and correlates with levels of angiogenic cytokines and bone marrow microvascular density [42]. Although none of these functional results survived after correction for multiple testing and therefore need to be replicated, they are consistent with previous studies suggesting a relevant role of the *CD46* locus in MM pathogenesis. Considering that the *CD46*_rs1142469_ SNP also alters binding sites for Hoxa7 [43], a transcription factor that is frequently dysregulated in MM patients and plays a role in modulating hematopoiesis and cell differentiation [44], it seems conceivable to suggest that the *CD46*_rs1142469_ SNP could influence the risk of developing MM by modulating not only CD46 expression, but also the number of specific subsets of B and T cells and IL20^−^ and Hoxa7-mediated signaling pathways.

We also found, for the first time, that carriers of the *IKBKE*_rs17433804C_ allele had an increased risk of developing MM. Two additional neighboring SNPs in the same LD block confirmed the association of *IKBKE* SNPs with disease risk. The *IKBKE* locus is located at chromosome 1q32.1 and it acts as a modulator of multiple immune processes [45] including the necrosis factor (NF)-κB non-canonical pathway [46], a key process in MM pathogenesis. *IKBKE* expression is almost restricted to lymphoid tissue and Westra et al. reported that the *IKBKE*_rs17433804_ SNP is an eQTL marker for *IKBKE* in peripheral blood (*p* = 9.65 × 10^−9^) [43]. In addition, it has been described that the *IKBKE* locus is frequently mutated in MM patients [47], which reinforces the idea of a role of this gene in determining MM risk. Interestingly, we also found that carriers of the *IKBKE*_rs17433804C_ allele had increased numbers of transitional CD24 + CD38+ B cells, which is a type of B cell linked to MM tumorigenesis. It has been reported that CD24+ cells are upregulated in MM and they have an increased expression of CXCR4 [48], which is a well-known chemokine receptor that plays a pivotal role in proliferation, invasion, dissemination, and drug resistance in MM [49]. However, CD24 expression has been also found to be associated to a more apoptotic and less tumorigenic phenotype by impaired capability to migrate and to create colonies as compared with CD24^−^ MM cells [48]. These controversial results might indicate that CD24 might have a dual effect on MM by contributing to disease onset but also by preventing disease progression. In line with this hypothesis, Gross Even-Zohar et al. reported that patients having high expression of CD24^+^ MM cells had longer overall survival and progression free survival [50]. In addition, we found that carriers of the *IKBKE*_rs17433804C_ allele had decreased circulating serum concentrations of MCP-2, which has been linked to chemotaxis of myeloid and lymphoid cells, but also to other important processes such as autophagy, leukocyte behavior, cell adhesion and polarization, cell secretion, and cell survival of MM cells [51]. A recent study demonstrated that MCP-1, another chemokine from the MCP family, was increased in MM patients and correlated with clinical characteristics and enhanced angiogenesis in MM [52]. In line with these results, another study demonstrated that the inhibition of MCP-2, as well as its receptor CCR2, by neutralizing antibodies reduced migration of MM cells [53]. Altogether, these findings suggest that the *IKBKE* locus might influence MM risk by modulating absolute numbers of specific subsets of B-cells and MCP-2-mediated immune responses and MM cell migration. Nonetheless, it is also important to consider that the presence of this variant alters regulatory motifs for multiple transcription factors including AP-1, p300, TCF4, Myc, and PRMD1 [43]. These transcription factors which are highly expressed in MM [54,55] have been involved in B cell development and plasma cell differentiation, MM cell proliferation, cell survival, and drug resistance in the bone marrow microenvironment [56,57,58], and they even exhibit antitumoral activity in MM pathogenesis [54,59]. Even though the above reported results shed some light on the role of the *IKBKE* locus in MM, additional functional studies are still warranted to establish the exact mechanism by which the *IKBKE* locus is linked to MM onset and disease progression.

Another novel finding was the association between the *PARK2*_rs1884158_ SNP (and four neighboring SNPs) and the risk of MM. In addition to its role in autophagy, *PARK2* is involved in modulating stress response, mitochondrial biogenesis, stability of G1/S cyclins [60], cell growth (acting as tumor suppressor), and mitophagy, where it purges damaged organelles from the vital mitochondrial network [61]. Although the *PARK2* gene was associated with the risk of solid tumors [62], little is known about its role in MM. Our functional experiments did not show any positive correlation between the *PARK2*_rs1884158_ SNP and host immune responses and, to the best of our knowledge, no functional roles for this SNP have been reported in Haploreg or eQTL browsers. Therefore, additional studies are still warranted to replicate the association of the *PARK2*_rs1884158_ SNP with MM risk and, subsequently, to characterize its functional role in MM pathogenesis.

As well as the novel association of *CD46*, *IKBKE*, and *PARK2* SNPs with MM risk, we could validate the association of SNPs within well-known MM susceptibility genes such as *ULK4*, *ATG5*, and *CDKN2A* [39,63]. The strongest association was found for the *ULK4*_rs6599175_ polymorphism within the *ULK4* gene. Each copy of the *ULK4*_rs6599175C_ allele increased the risk of developing MM by 31%. This result agreed with a previous study by Broderick et al. (2011) [63]. The *ULK4* gene is located at chromosome 3p22.1 and encodes for a member of the unc-51-like serine/threonine kinase (STK) family that plays a role in modulating hypertension [64] and cardiologic disorders [65]. According to GTEx portal data and other eQTL studies [40], the *ULK4*_rs6599175C_ allele strongly correlates with higher levels of *ULK4* mRNA expression levels in multiple tissues including whole blood (*p* = 1.4 × 10^−59^). However, outside of mRNA expression levels, little is known about the functional role of this genetic variant to modulate the risk of MM. In this regard, we found that carriers of the *ULK4*_rs6599175C_ risk allele correlated with increased serum concentrations of vitamin D3 in healthy donors, which might suggest that the effect of the *ULK4*_rs6599175C_ allele to increase the risk of MM might be mediated by a vitamin D-dependent mechanism likely affecting cell differentiation and proliferation, angiogenesis, immune responses, and apoptosis either in normal or malignant tissues [66,67,68]. In addition, this SNP has been linked to monoclonal gammopathy of undetermined significance (MGUS), a non-cancerous precursor condition that precedes MM [69], suggesting that the *ULK4*_rs6599175_ SNP might exert its biological effect during the earliest stages of plasma cell dyscrasia progression from early to late-stage disease. Additional studies are required to decipher the interplay between the *ULK4* locus and circulating vitamin D3 levels in the context of MM risk and progression.

On the other hand, the *ATG5* is located on chromosome 6q21 and it has been related to autophagy vesicle formation and apoptosis [70]. A previous study reported the association between the *ATG5*_rs9372120_ SNP (a variant in strong LD with the rs2299864) and MM risk [39], but also MGUS [69], which suggests that the *ATG5* locus might also exert its biological effect on the risk of developing MM by acting at early and non-neoplastic stages. Although we could not find any correlation between the *ATG5*_rs2299864_ SNP and host immune responses or autophagy flux, it was reported that this variant is located among histone marks for primary T helper naive and memory cells and primary T CD8^+^ naive cells from peripheral blood and that it disrupts regulatory motifs for several transcription factors including DMRT3, which has an altered methylation profile during early stages of myelomagenesis [71].

Finally, the *CDKN2A* gene is located on chromosome 9p21.3 and encodes for a cyclin-dependent kinase inhibitor 2A that has been involved in p53 stabilization, cell cycle arrest, and cell proliferation. Unlike the *ULK4*_rs6599175_ or *ATG5*_rs2299864_ SNPs, the *CDKN2A*_rs2811710_ SNP has not been linked to MGUS susceptibility, which suggests that the *CDKN2A*_rs2811710_ SNP might be involved in promoting the accumulation of tumorigenic MM cells rather than acting in earlier stages. In fact, several germline mutations within the *CDKN2A* have been consistently associated with MM predisposition [72], but also improved patient overall survival [73]. Interestingly, we found that carriers of the *CDKN2A*_rs2811710C_ allele, which is associated with an increased risk of MM, had decreased numbers of CD4^+^EMCD45RO^+^ CD27^−^ T memory cells, which are cells that display a high antigen recall response and were reported to be poorly represented in MM patients compared to controls [74]. In addition, the *CDKN2A*_rs2811710_ SNP was associated with the expression of *CDKN2B* and *STK16* mRNAs in monocytes that are involved in the modulation of cell cycle arrest through the TFG signaling pathway [75]. The *CDKN2A*_rs2811710_ SNP maps among histone marks for primary T helper memory cells and primary T regulatory cells from peripheral blood and alters binding sites for key transcription factors such as HDAC2, HNF1, OTX, and p300 that play important roles in epigenetic repression and transcriptional regulation, cell cycle progression, and developmental events, and are even involved in influencing overall survival [76].

It is worth mentioning that this study has both strengths and limitations. The major strengths of our study were the comprehensive analysis of autophagy-related SNPs and the inclusion of three large independent European populations of European ancestry including a total of 12,676 participants. Furthermore, we comprehensively analyzed the impact of autophagy-related SNPs in modulating blood cell counts, steroid hormones, serum and plasma metabolites, and immune responses in a large study of healthy subjects ascertained through the HFGP. Another important strength of this study was the experimental analysis assessing the effect of autophagy SNPs in modulating the autophagy flux in PBMCs left untreated or treated with metformin or bafilomycin. This approach allowed us to investigate, for the first time, the impact of susceptibility loci for MM on the autophagy flux. A limitation of this study was its multicentric nature that placed inevitable limitations such as the impossibility of uniformly collected mutation and/or cytogenetic profiles for a significant proportion of patients analyzed. In addition, this study included a subset of German participants, which overlapped with the GWAS used for discovering the ULK4 MM susceptibility locus. Finally, given that all study participants included in this study were of European ancestry, we could not determine the impact of autophagy variants in other ethnic or ancestral populations. Additional studies using other ethnic and/or ancestry populations are now warranted to confirm the results described herein.

## 4. Materials and Methods

**Figure 4:**
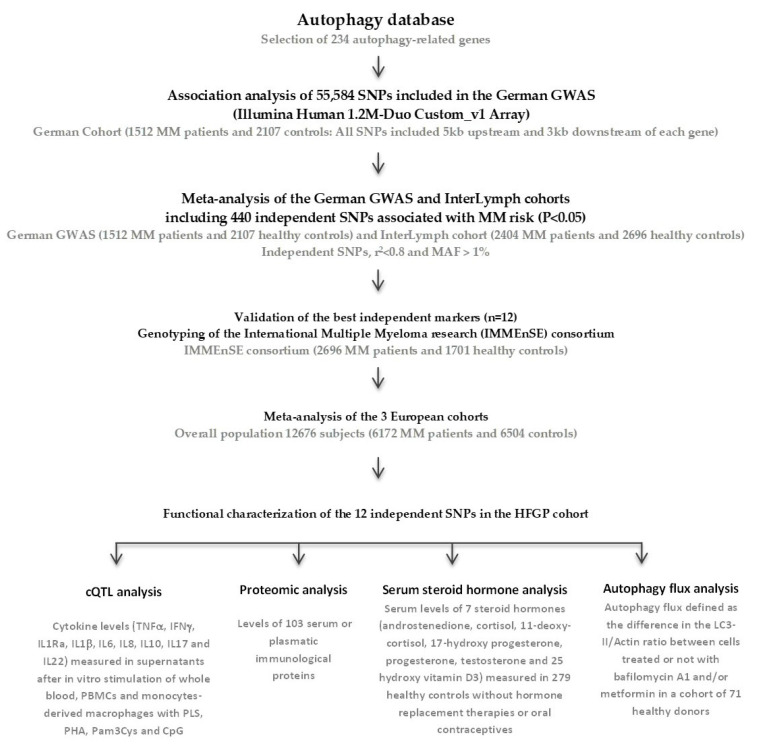
Flow diagram of the study.

A workflow diagram of the study is included in Figure 4.

### 4.1. Study Populations

The discovery population consisted of 3169 subjects from GWAS conducted by the German-Speaking Multiple Myeloma Multicenter Study Group (GMMG), followed by meta-analysis with an independent GWAS on 5100 subjects by the International Lymphoma Epidemiology Consortium (InterLymph; https://epi.grants.cancer.gov/interlymph/; accessed on 12 February 2020). International Myeloma Working Group (IMWG) criteria were used by physicians to establish the diagnosis of MM [77,78]. Ethical approval for these respective studies was obtained from each participating institution and all participanting subjects provided written informed consent. The German GWAS coordinated by the University Clinic, Heidelberg (ISRCTN06413384: GMMG-HD3 http://www.isrctn.com/search?q=ISRCTN06413384, accessed on 12 February 2020; ISRCTN64455289: GMMG-HD4 http://www.isrctn.com/search?q=ISRCTN64455289, accessed on 12 February 2020; and ISRCTN05745813: GMMG-HD5 http://www.isrctn.com/search?q=ISRCTN05745813, accessed on 12 February 2020) included 1512 MM patients that were genotyped using Illumina Human OmniExpress-12 v1.0 arrays and 2107 healthy individuals enrolled into the Heinz Nixdorf Recall (HNR) study that were genotyped using either the Illumina HumanOmni1-Quad_v1 or OmniExpress-12 v1.0 arrays [39]. The InterLymph MM GWAS included 2404 MM and 2696 controls that were genotyped using Affymetrix, Human660W-quad Beadchip, and Illumina arrays 610 Quad, Omni5, OmniExpress Beadchip, and OncoArray [69]. Both German and InterLymph GWAS were independently subjected to rigorous standard quality controls prior to imputation using the IMPUTE2 v2.3 software or the Michigan imputation server (based on the Haplotype Reference Consortium), respectively [79]. After imputation, each site filtered the data to include only high-quality imputed variants (information score > 0.8), and further quality-control checks were implemented including checks for missingness, duplicates, abnormal heterozygosity, cryptic relatedness, population outliers (evaluated by principal components analyses using Eigenstrat software), and genomic inflation (λ = 1.00) [39,69].

### 4.2. SNP Selection

A total of 234 autophagy-related genes were selected on the basis of their presence in the autophagy database (http://autophagy.lu/index.html, accessed on 12 February 2020; Supplementary Table S1). Estimates of association with MM risk for all genotyped or imputed SNPs within or near these genes (5 Kb up-stream and 3 Kb downstream) were extracted from the German GWAS [39]. After testing Hardy-Weinberg Equilibrium (HWE) in the control group by a standard observed-expected chi-square (χ^2^) test (*p* < 10^−5^), 3018 SNPs were selected according to their level of association with MM risk (*p* ≤ 0.05), but also considering linkage disequilibrium values (r^2^ < 0.8) and minor allele frequency (MAF) of > 0.01. Among them, 440 SNPs were independent (r^2^ < 0.1) according to LDLink information (https://ldlink.nci.nih.gov/?tab=snpclip, accessed on 12 February 2020) and, therefore, the multiple testing significance threshold was set to 1.14 × 10^−4^ (0.05/440 independent SNPs; Supplementary Table S2).

### 4.3. Replication Cohort, Genotyping, and Meta-Analysis

Following the same approach, these SNPs were then tested for association with MM risk in the InterLymph MM GWAS and a meta-analysis of both study populations was conducted using METAL [80]. The I^2^ statistic was used to assess statistical heterogeneity between the studies and the pooled odds ratio (OR) was computed using the fixed-effect model for SNPs showing non-significant heterogeneity and the random-effect model for SNPs reporting significant heterogeneity. After the meta-analysis of both populations, 12 SNPs were advanced for replication in the International Multiple Myeloma (IMMEnSE) consortium, consisting of 2696 MM patients and 1701 controls. As for the GWAS populations, the Ethical Committee of the Faculty of Medicine of the University of Heidelberg approved the IMMEnSE study protocol and written informed consent from each study participant was obtained [81].

Genotyping of selected SNPs in the IMMEnSE study was carried out at DKFZ (German Cancer Research Center, Heidelberg, Germany) using KASPar^®^ (LGC Genomics, Hoddesdon, UK) or Taqman^®^ SNP Genotyping assays (Thermo Fisher Scientific, Foster City, CA, USA) according to previously reported protocols [82]. For internal quality control, ~5% of samples were randomly selected and included as duplicates. Concordance between the original and the duplicate samples for the 12 SNPs tested was ≥99.0%. All SNPs showed genotype frequencies in the control population similar to those found in the 1000 Genomes database and were in HWE.

### 4.4. Functional Effect of the Autophagy-Related Variants on Immune Responses

To provide insight into the functional role of the most interesting autophagy SNPs in modulating immune responses, we tested if any of these genetic markers correlated with cytokine expression quantitative trait loci (cQTL) data from in vitro stimulation experiments, but also absolute numbers of 91 blood-derived cell populations and 103 serum or plasmatic inflammatory proteins quantified in the approximately 500 volunteers from the 500 Functional Genomics cohort from the Human Functional Genomics Project (HFGP; http://www.humanfunctionalgenomics.org/site/, accessed on 12 February 2020). The HFGP study was approved by the Arnhem-Nijmegen Ethical Committee (no. 42561.091.12) and biological specimens were collected after informed consent was obtained.

cQTL data included cytokine levels (IFNγ, IL1β, IL6, TNFα, IL17, and IL22) after the stimulation of peripheral blood mononuclear cells (PBMCs), monocyte-derived macrophages (MDM), or whole blood from 408 healthy subjects with LPS (1 or 100 ng/mL; Sigma Aldrich, St. Louis, MO, USA), PHA (10 µg/mL, Sigma, St. Louis, MO, USA), Pam3Cys (10 µg/mL, EMC microcollections, Tübingen, Germany), or CpG (100 ng/mL, InvivoGen, San Diego, CA, USA). Concentrations of human IFNγ, IL1β, IL6, TNFα, IL17, and IL22 were determined using specific commercial ELISA kits (PeliKine Compact, Amsterdam, or R&D Systems), in accordance with the manufacturers’ instructions. When values were below or above the detection limit of the ELISA, the corresponding limit was used. After log transformation, linear regression analyses adjusted for age and sex were used to determine the correlation of the selected SNPs with cQTL data. Details on PBMC isolation, macrophage differentiation, and stimulation assays have been reported elsewhere [83].

To evaluate the impact of autophagy SNPs on cell-level variation, 91 blood-derived cell populations were measured by 10-color flow cytometry (Navios flow cytometer, Beckman Coulter, Miami, FL, USA) after blood sampling (2–3 h), and cell count analysis was performed using Kaluza software (Beckman Coulter, v.1.3). In order to reduce inter-experimental noise and increase statistical power, cell count analysis was performed by calculating parental and grandparental percentages, which were defined as the percentage of a certain cell type within the subpopulation of the cells from which it was isolated [48]. Detailed laboratory protocols for cell isolation, reagents, gating, and flow cytometry analysis have been reported elsewhere [84]. The accession number for the raw flow cytometry data and analyzed data files are available upon request to the authors (accessed on 13 December 2019; Supplementary Table S3).

A proteomic analysis was also performed in serum and plasma samples from the HFGP study. Circulating proteins were measured using the commercial Olink^®^ Inflammation panel (Olink, Sweden) that resulted in the measurement of 103 different biomarkers (Supplementary Table S4). Protein levels were expressed on a log2-scale as normalized protein expression values and normalized using bridging samples to correct for batch variation.

All analyses were performed using R software (http://www.r-project.org/, accessed on 12 February 2020), using custom scripts in the R programming language based on existing functions such as lm (stats). In order to account for multiple comparisons, we used Bonferroni corrected significant thresholds of 0.00069 (0.05/12 SNPs/6 cytokines), 4.57 × 10^−5^ (0.05/12 SNPs/91 cell-derived populations), and 4.05 × 10^−5^ (0.05/12 SNPs/103 proteins) for the cQTL, cell variability, and proteomic analyses, respectively.

### 4.5. Correlation between Autophagy-Related SNPs and Steroid Hormone Levels

In addition to the immunological data, we also assessed the association of autophagy SNPs with data on steroid hormone levels (androstenedione, cortisol, 11-deoxy-cortisol, 17-hydroxy progesterone, progesterone, testosterone, and 25 hydroxy vitamin D3) from 279 healthy controls of the HFGP study without hormone replacement or oral contraceptive therapies. Serum steroid hormone levels were determined by ELISA following the manufacturer’s instructions. Correlation between levels of seven serum steroid hormones and autophagy-related SNPs was evaluated by linear regression analysis adjusted for age and sex. The significance threshold was set to *p* = 7.1 × 10^−4^, considering the number of independent SNPs (*n* = 12) and the number of hormones tested (*n* = 7).

### 4.6. Impact of Autophagy-Related Variants on the Autophagy Flux

In order to accurately determine the role of autophagy SNPs in modulating autophagy, we investigated their impact on the autophagy flux in a population of 68 European healthy donors. For that purpose, we isolated peripheral blood mononuclear cells (PBMCs) from whole blood by density gradient centrifugation using Histopaque^®^, and we treated them for 2 h with 10 µM of bafilomycin A1 or 10 mM of metformin to inhibit or induce autophagy, respectively. A total of 5 × 10^5^ PBMCs were plated in each well for stimulatory and inhibitory experiments and treated with metformin or bafilomycin A1 alone or in combination. Untreated cells were used as experimental controls. After treatment, cells were harvested and protein extraction was performed with 50 µL of lysis buffer (1% NP-40, 500 mM Tris HCL, 2.5 M NaCl, 20 mM EDTA, phosphatase and protease inhibitors—from Roche—at pH 7.2). Then 20 µg of the total protein were resolved in a 12% SDS gel and transferred to a nitrocellulose membrane for 10 min in a Trans-Blot Turbo transfer system. Membranes were then blocked for 1 h using Tris-buffered saline (TBS) with 0.1% Tween 20 (TBST) containing 5% BSA and incubated overnight at 4 °C with the polyclonal primary antibodies at 1:1000 in 1% BSA (Rabbit anti-LC3A/B Antibody, Cell-Signaling and Mouse anti-Actin antibody, Merck Millipore, Darmstadt, Germany). After washing with tris-buffered saline-tween (TBS-T), nitrocellulose membranes were incubated with the corresponding secondary antibodies (IgG anti-Rabbit for LC3A/B and IgG anti-Mouse for Actin). Protein levels were detected after incubation with SuperSignal West Femto Maximum Sensitivity Substrate (Thermofisher, Waltham, MA, USA) or Clarity Western ECL Substrate (Bio-Rad, Hercules, CA, USA). Digital images of the Western blots were obtained in a ChemiDoc XRS System (Bio-Rad) with Quantity One software V4.6.5 (Bio-Rad). Autophagy flux was determined as the difference in the LC3-II/Actin ratio between cells treated or not with bafilomycin A1 and/or metformin. Linear regression analyses adjusted for age and sex were used to determine the correlation between autophagy-related SNPs and autophagy flux values. A significance threshold of *p* = 0.0028 was set according to the number of SNPs tested (*n* = 12) and the treatments administrated in vitro (*n* = 2).

### 4.7. In Silico Functional Analysis

Haploreg (http://www.broadinstitute.org/mammals/haploreg/haploreg.php, accessed on 12 February 2020) and ENCODE annotation data (https://genome.ucsc.edu/ENCODE) were also used to predict the functional role of the most interesting autophagy SNPs. We also analyzed whether selected SNPs could be expression quantitative loci (eQTL) for different cell types and tissues using data from public eQTL browsers such as GTex portal (https://gtexportal.org/home/, accessed on 12 February 2020), Blood eQTL browser (https://genenetwork.nl/bloodeqtlbrowser/, accessed on 12 February 2020), and Haploreg [43].

## 5. Conclusions

This study identifies novel associations for genetic polymorphisms within the *CD46*, *IKBKE*, and *PARK2* loci and MM risk and validates previously reported associations for SNPs within the *ULK4*, *ATG5*, and *CDKN2A* genes. Although the biological effect of the *CD46*, *IKBKE*, *ULK4*, and *CDKN2A* SNPs seemed to be mediated by alterations of absolute numbers of certain B- and T-cell subsets and MCP-2-, IL20-, and vitamin D3-dependent mechanisms affecting cell differentiation and proliferation, angiogenesis, immune responses, and apoptosis, we could not identify the biological mechanisms underscoring the *ATG5* association.

## Figures and Tables

**Table 1:** Association analysis of autophagy-related SNPs in the discovery cohorts.

			German GWAS (*n* = 3619)		InterLymph GWAS (*n* = 5100)		Meta-Analysis (*n* = 8719)		
SNP	Gene	A1	OR (95%CI)	*p*	OR (95%CI)	*p*	OR (95%CI)	*p*	*P_Het_*
rs2299864	*ATG5*	T	1.18 (1.03–1.33)	0.015	1.24 (1.12–1.36)	1.96 × 10^−5^	1.22 (1.13–1.32)	1.48 × 10^−6^	0.545
rs1142469	*CD46*	A	1.14 (1.02–1.25)	0.020	1.11 (1.02–1.21)	0.012	1.12 (1.05–1.20)	9.68 × 10^−4^	0.694
rs2811710	*CDKN2A*	C	1.15 (1.03–1.29)	0.011	1.11 (1.02–1.20)	0.0176	1.12 (1.05–1.20)	5.28 × 10^−4^	0.623
rs143309009	*CTSD*	G	2.22 (1.46–3.38)	2.1 × 10^−4^	1.24 (0.89–1.72)	0.2114	1.55 (1.20–2.01)	9.14 × 10^−4^	0.032
rs11064698	*HSPB8*	T	1.53 (1.05–2.24)	0.027	1.16 (1.06–1.27)	0.0012	1.18 (1.08–1.28)	2.46 × 10^−4^	0.163
rs12739461	*IKBKE*	T	1.18 (1.05–1.32)	0.0053	1.10 (1.01–1.20)	0.028	1.13 (1.05–1.21)	6.36 × 10^−4^	0.337
rs2297546	*IKBKE*	G	1.22 (1.09–1.37)	7.2 × 10^−4^	1.09 (1.01–1.18)	0.037	1.13 (1.06–1.21)	2.25 × 10^−4^	0.110
rs17433804	*IKBKE*	C	1.24 (1.09–1.41)	0.0011	1.08 (1.00–1.18)	0.066	1.13 (1.05–1.21)	7.51 × 10^−4^	0.086
rs1884158	*PARK2*	T	1.21 (1.08–1.35)	0.00081	1.09 (1.00–1.18)	0.049	1.14 (1.06–1.21)	3.07 × 10^−4^	0.141
rs34048269	*RPTOR*	A	1.30 (1.14–1.48)	1.3 × 10^−4^	1.06 (0.97–1.17)	0.199	1.14 (1.05–1.23)	0.0024	0.013
rs6599175	*ULK4*	C	1.33 (1.16–1.53)	5.7 × 10^−5^	1.25 (1.13–1.39)	3.6 × 10^−5^	1.28 (1.18–1.39)	4.00 × 10^−9^	0.482
rs7202154	*USP10*	G	1.31 (1.06–1.62)	0.011	1.23 (1.05–1.44)	0.0126	1.26 (1.11–1.43)	3.83 × 10^−4^	0.640

Abbreviations: SNP, single nucleotide polymorphism; A1, effect-allele.

**Table 2:** Association estimates of the 12 autophagy SNPs representing susceptibility regions for MM.

	German GWAS (*n* = 3619)		InterLymph GWAS (*n* = 5100)		IMMEnSE (*n* = 3957)		Meta-Analysis (*n* = 12676)		
Gene_SNP	OR (95%CI)	*p*	OR (95%CI)	*p*	OR (95%CI)	*p*	OR (95%CI)	*P*_Meta_	*P_Het_*
*ATG5_*_rs2299864T_	1.18 (1.03–1.33)	0.015	1.24 (1.12–1.36)	1.96 × 10^−5^	1.07 (0.92–1.23)	0.40	1.18 (1.11–1.27)	1.3 × 10^−6^ Ϯ	0.254
*CD46_*_rs1142469A_	1.14 (1.02–1.25)	0.020	1.11 (1.02–1.21)	0.012	1.09 (0.96–1.23)	0.19	1.12 (1.05–1.18)	2.2 × 10^−4^	0.851
*CDKN2A_*_rs2811710C_	1.15 (1.03–1.29)	0.011	1.11 (1.02–1.20)	0.0176	1.18 (1.06–1.32)	0.003	1.14 (1.08–1.20)	7.0 × 10^−6^ Ϯ	0.666
*CTSD_*_rs143309009G_	2.22 (1.46–3.38)	2.1 × 10^−4^	1.24 (0.89–1.72)	0.2114	0.72 (0.47–1.10)	0.13	1.26 (1.01–1.57)	0.042	0.001
*HSPB8_*_rs11064698T_	1.53 (1.05–2.24)	0.027	1.16 (1.06–1.27)	0.0012	0.84 (0.72–0.99)	0.035	1.09 (1.01–1.18)	0.029	0.001
*IKBKE_*_rs12739461T_	1.18 (1.05–1.32)	0.0053	1.10 (1.01–1.20)	0.028	1.01 (0.90–1.14)	0.88	1.10 (1.03–1.16)	0.0022	0.179
*IKBKE_*_rs2297546G_	1.22 (1.09–1.37)	7.2 × 10^−4^	1.09 (1.01–1.18)	0.037	1.00 (0.90–1.12)	0.94	1.10 (1.04–1.16)	0.0013	0.047
*IKBKE_*_rs17433804C_	1.24 (1.09–1.41)	0.0011	1.08 (1.00–1.18)	0.066	1.16 (1.02–1.31)	0.024	1.14 (1.07–1.21)	4.6 × 10^−5^ Ϯ	0.213
*PARK2_*_rs1884158T_	1.21 (1.08–1.35)	0.00081	1.09 (1.00–1.18)	0.049	1.04 (0.91–1.19)	0.56	1.11 (1.05–1.18)	4.5 × 10^−4^	0.184
*RPTOR___*_rs34048269A_	1.30 (1.14–1.48)	1.3 × 10^−4^	1.06 (0.97–1.17)	0.199	1.04 (0.91–1.19)	0.56	1.11 (1.04–1.19)	0.0017	0.024
*ULK4___*_rs6599175C_	1.33 (1.16–1.53)	5.7 × 10^−5^	1.25 (1.13–1.39)	3.6 × 10^−5^	1.37 (1.21–1.56)	2.6 × 10^−6^	1.31 (1.22–1.40)	5.8 × 10^−14^ Ϯ	0.522
*USP10*__rs7202154G_	1.31 (1.06–1.62)	0.011	1.23 (1.05–1.44)	0.0126	0.93 (0.76–1.15)	0.50	1.16 (1.04–1.29)	0.0075	0.048

Abbreviations: SNP, single nucleotide polymorphism; A1, effect allele; Ϯ Significant after correction for multiple testing.

## Data Availability

The genotype data used in the present study are available by the corresponding authors upon reasonable request. Functional data used in this project have been meticulously catalogued and archived in the BBMRI-NL data infrastructure (https://hfgp.bbmri.nl/, accessed on 12 February 2020) using the MOLGENIS open-source platform for scientific data. This allows flexible data querying and download, including sufficiently rich metadata and interfaces for machine processing (R statistics, REST API) and using FAIR principles to optimize Findability, Accessibility, Interoperability, and Reusability.

## Supplementary Materials

The following supporting information can be downloaded at: https://www.mdpi.com/article/10.3390/ijms24108500/s1.
