# Supplementary material for: Polymorphisms within Autophagy-Related Genes as Susceptibility Biomarkers for Multiple Myeloma: A Meta-Analysis of Three Large Cohorts and Functional Characterization
Source: Int J Mol Sci. 2023 May 9;24(10):8500. doi: 10.3390/ijms24108500 (PMC10218542; doi:10.3390/ijms24108500)
Supplement: Supplementary file 1 [file ijms-24-08500-s001.zip › Sanchez-Maldonado_JM[1]. Supplementary Figure 1_Locus Zoom.pdf]

Supplementary Figure 1. Locus Zoom plots of the autophagy-related SNPs representing susceptibility regions for MM (after meta-analysis of the German and InterLymph populations).

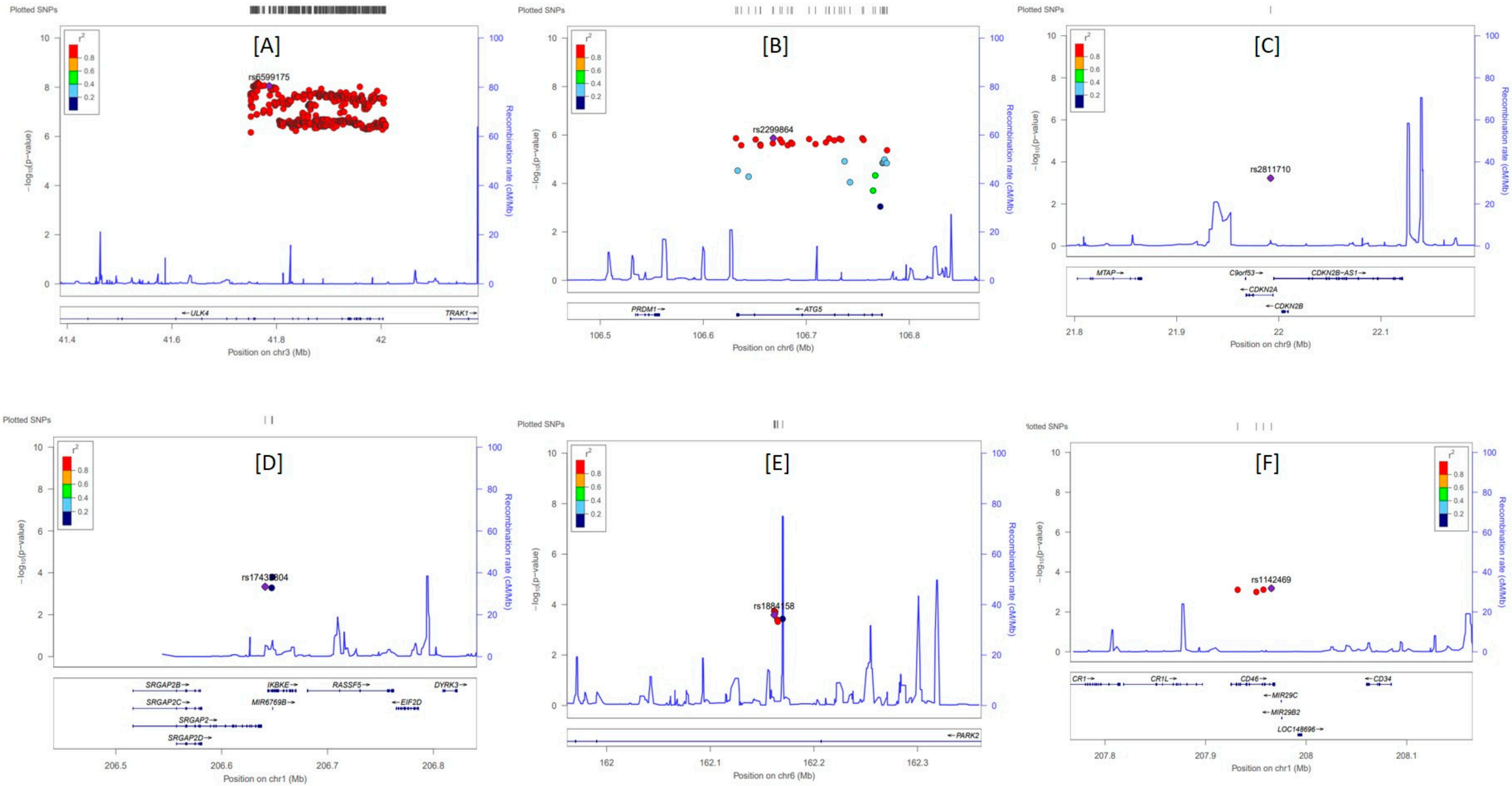

[A] LocusZoom plot of the *ULK4* gene in chromosome 3 than includes the rs6599175 SNP (P=8.94·10^{-09}) but also 501 additional SNPs associated with MM risk; **[B]** LocusZoom plot of the *ATG5* gene including the rs2299864 SNP (P= 1.35·10^{-06}) but also 35 additional markers associated with MM risk; **[C]** LocusZoom plot of the *CDKN2A* gene including the rs2811710 SNP (P= 5.93·10^{-04}). Although only this SNP in the *CDKN2A* locus was available in the GWAS datasets, this marker has been consistently associated with MM risk; **[D]** LocusZoom plot of the *IKBKE* gene encompassing the rs17433804 SNP in chromosome 1 (p= 4.62·10^{-04}) but also 2 neighboring SNPs showing a similar level of association. **[E]** LocusZoom plot of the *CD46* gene including the rs1142469 SNP (p= 6.55·10^{-04}) and 3 neighboring SNPs; **[F]** LocusZoom plot of the *PARK2* gene region encompassing the rs1884158 SNP (p= 2.56·10^{-04}) and 4 additional neighboring SNPs. All the results are represented according to hg19 genome build.
