# Supplementary material for: Polymorphisms within Autophagy-Related Genes as Susceptibility Biomarkers for Multiple Myeloma: A Meta-Analysis of Three Large Cohorts and Functional Characterization
Source: Int J Mol Sci. 2023 May 9;24(10):8500. doi: 10.3390/ijms24108500 (PMC10218542; doi:10.3390/ijms24108500)
Supplement: Supplementary file 1 [file ijms-24-08500-s001.zip › Sanchez-Maldonado_JM[1]. Supplementary Table_2_SNP_list.pdf]

Supplementary Table 2. Selected autophagy-related SNPs.

Chr	Position (GRCh37)	dbSNP rs#	Nucleotide substitution	Amino acid substitution	Gene	MAF (CEU)
1	3595768	rs10910008	C/G	intronic	TP73	C=0.618
1	161841018	rs112462458	G/T	intronic	ATF6	G=0.981
1	242159710	rs114367814	G/T	intronic	MAP1LC3C	G=0.939
1	110133967	rs116212438	T/C	intronic	GNAI3	T=0.989
1	87173249	rs12409329	T/C	intronic	SH3GLB1	T=0.933
1	223901842	rs142942507	C/G	intronic	CAPN2	C=0.983
1	226587507	rs143161050	T/C	intronic	PARP1	T=0.984
1	10551365	rs147964123	C/G	intronic	PEX14	C=0.980
1	161853671	rs148690272	G/T	intronic	ATF6	G=0.980
1	3639884	rs149926186	T/C	intronic	TP73	T=0.986
1	63284830	rs17123872	G/A	exonic	ATG4C	G=0972
1	11273213	rs17374795	A/G	intronic	MTOR	A=0.978
1	206641052	rs17433804	C/T	intergenic	SRGAP2,IKBKE	C=0.689
1	206648037	rs2297546	C/G	intronic	IKBKE	C=0.600
1	206647218	rs12739461	A/G	UTR5	IKBKE	A=0.753
1	87207872	rs263478	G/T	intronic	SH3GLB1	G=0.862
1	207965427	rs1142469	G/A	intronic	CD46	A=0.845
1	226569987	rs3219069	G/A	intronic	PARP1	G=0.975
1	207931996	rs35677203	A/T	intronic	CD46	A=0.926
1	161765834	rs35699192	G/A	intronic	ATF6	G=0.986
1	160183740	rs41265779	C/T	UTR3	PEA15	C=0.969
1	206665240	rs41299852	T/C	intronic	IKBKE	T=0.972
1	87170417	rs58494650	C/T	UTR5	SH3GLB1	C=0.972
1	20956912	rs61781981	G/A	intergenic	CDA,PINK1	G=0.943
1	87169118	rs61802920	T/C	intergenic	CLCA3P,SH3GLB1	T=0.908
1	3572688	rs6663736	T/C	intronic	TP73	T=0.698
1	87173963	rs74871690	T/C	intronic	SH3GLB1	T=0.972

1	10615982	rs78432262	G/A	intronic	PEX14	G=0.947
1	11185739	rs79373174	G/A	intronic	MTOR	G=0.899
1	10568140	rs79776554	C/T	intronic	PEX14	C=0.986
1	160183100	rs8175359	G/A	intronic	PEA15	G=0.974
2	183862903	rs115885826	C/T	intronic	NCKAP1	C=0.981
2	37343771	rs116285413	A/G	intronic	EIF2AK2	A=0.988
2	32663128	rs147477721	A/G	intronic	BIRC6	A=0.981
2	32610642	rs147749755	A/G	intronic	BIRC6	A=0.989
2	32646160	rs151116561	C/G	intronic	BIRC6	C=0.987
2	173321021	rs16860435	A/G	intronic	ITGA6	A=0.943
2	136873549	rs2680880	A/T	UTR5	CXCR4	A=0.582
2	183856033	rs34565726	C/T	intronic	NCKAP1	C=0.985
2	32603640	rs4019436	C/G	intronic	BIRC6	C=0.691
2	136880385	rs59222832	T/C	intergenic	CXCR4,THSD7B	T=0.883
2	32592318	rs62136267	G/T	intronic	BIRC6	G=0.821
2	32605698	rs6748386	A/G	intronic	BIRC6	G=0.946
2	37339084	rs7577041	A/C	intronic	EIF2AK2	A=0.735
3	189395209	rs10049471	G/A	intronic	TP63	G=0.567
3	189399069	rs111534976	A/G	intronic	TP63	A=0.951
3	41942795	rs112132603	C/T	intronic	ULK4	C=0.982
3	41417969	rs112607713	C/T	intronic	ULK4	C=0.937
3	11372520	rs114851724	A/G	intronic	ATG7	A=0.977
3	5227522	rs115578554	G/A	intergenic	ARL8B,EDEM1	G=0.977
3	4552969	rs115769448	A/T	intronic	ITPR1	A=0.987
3	172240458	rs115861969	A/G	intronic	TNFSF10	A=0.981
3	11570026	rs116166360	C/T	intronic	ATG7	C=0.982
3	189498124	rs12486980	C/T	intronic	TP63	C=0.902
3	128522922	rs12493061	A/C	intronic	RAB7A	A=0.971
3	189495337	rs13095322	G/A	intronic	TP63	G=0.901
3	11325549	rs138578039	C/T	intronic	ATG7	C=0.979

3	41368080	rs139438255	G/A	intronic	ULK4	G=0.982
3	183390462	rs139710544	G/A	intronic	KLHL24	G=0.966
3	189608067	rs142526070	C/T	intronic	TP63	C=0.978
3	41302942	rs144700803	C/T	intronic	ULK4	C=0.978
3	87288213	rs146749265	G/C	intronic	CHMP2B	G=0.984
3	87279318	rs1488362	C/G	intronic	CHMP2B	C=0.552
3	41494653	rs149188609	C/A	intronic	ULK4	C=0.989
3	5239700	rs150377008	C/T	intronic	EDEM1	C=0.970
3	4637939	rs17041030	C/T	intronic	ITPR1	C=0.620
3	4793006	rs17041333	T/G	ncRNA_exonic	EGOT	T=0.983
3	4876515	rs17041517	G/A	intronic	ITPR1	A=0.506
3	172240215	rs1823227	T/G	intronic	TNFSF10	T=0.703
3	19991202	rs191668575	A/G	intronic	RAB5A	A=0.989
3	189567818	rs2056124	G/C	intronic	TP63	C=0.706
3	41312821	rs2371487	G/A	intronic	ULK4	G=0.889
3	4641530	rs2686621	C/T	intronic	ITPR1	T=0.950
3	189596662	rs2889923	T/C	intronic	TP63	T=0.985
3	46400062	rs3092960	G/A	exonic	CCR2	G=0.866
3	4580685	rs34253984	C/G	intronic	ITPR1	C=0.948
3	4714920	rs35789999	A/G	exonic	ITPR1	A=0.981
3	4717972	rs35826345	G/A	intronic	ITPR1	G=0.862
3	87276571	rs36098294	C/T	UTR5	CHMP2B	C=0.981
3	4743623	rs3792491	C/A	intronic	ITPR1	C=0.883
3	4628541	rs4685771	C/T	intronic	ITPR1	C=0.890
3	4877937	rs4685831	C/G	intronic	ITPR1	C=0.864
3	41829397	rs4973892	A/G	intronic	ULK4	A=0.546
3	4674710	rs56310584	C/G	intronic	ITPR1	C=0.932
3	41571253	rs62256860	G/A	intronic	ULK4	G=0.966
3	189509595	rs62279941	T/C	intronic	TP63	T=0.936
3	184052420	rs62287502	G/T	intronic	EIF4G1	G=0.966

3	4630987	rs6442889	G/A	intronic	ITPR1	G=0.877
3	189393745	rs6780531	A/G	intronic	TP63	G=0.759
3	4666434	rs6797564	C/G	intronic	ITPR1	C=0.966
3	19983631	rs71316239	C/T	intergenic	EFHB,RAB5A	C=0.839
3	41310016	rs73083808	G/A	intronic	ULK4	G=0.963
3	41786009	rs6599175	G/A	intronic	ULK4	G=0.969
3	189364425	rs75579144	G/A	intronic	TP63	G=0.957
3	189547735	rs75715827	T/C	ncRNA_exonic	MIR944	T=0.923
3	189406870	rs77953198	C/T	intronic	TP63	C=0.974
3	189353729	rs78392253	A/G	intronic	TP63	A=0.985
3	4691378	rs78619297	G/T	intronic	ITPR1	G=0.901
3	41571944	rs80350031	T/C	intronic	ULK4	T=0.976
3	189363575	rs9823045	C/G	intronic	TP63	G=0.516
3	189364890	rs9824378	T/G	intronic	TP63	G=0.880
3	41533596	rs9839278	A/C	intronic	ULK4	A=0.598
3	184038874	rs9846954	A/T	intronic	EIF4G1	A=0.751
3	4861852	rs9858749	A/G	intronic	ITPR1	G=0.935
3	41716317	rs994439	G/A	intronic	ULK4	A=0.514
4	94329816	rs114744335	A/G	intronic	GRID2	A=0.989
4	94583170	rs115503484	C/G	intronic	GRID2	C=0.988
4	94252722	rs11943480	A/C	intronic	GRID2	C=0.680
4	85869836	rs138984092	C/T	intronic	WDFY3	C=0.983
4	93375222	rs140568366	T/C	intronic	GRID2	T=0.985
4	85646032	rs144150483	T/A	intronic	WDFY3	T=0.986
4	93752534	rs17019918	A/G	intronic	GRID2	A=0.957
4	164056625	rs17574945	C/T	intronic	NAF1	C=0.938
4	93806504	rs190962045	A/C	intronic	GRID2	A=0.989
4	103536673	rs4648136	C/T	intronic	NFKB1	C=0.974
4	185575142	rs4862399	A/G	intronic	PRIMPOL	A=0.842
4	140371510	rs62323106	T/C	intergenic	NAA15,RAB33B	T=0.903

4	94447295	rs6819321	C/T	intronic	GRID2	C=0.559
4	93867037	rs71599264	C/T	intronic	GRID2	C=0.973
4	93259371	rs76688871	A/G	intronic	GRID2	A=0.973
4	93232542	rs79610780	C/T	intronic	GRID2	C=0.979
4	103442022	rs79843117	T/C	intronic	NFKB1	T=0.989
5	179694760	rs10058502	A/G	intronic	MAPK9	A=0.708
5	78106262	rs10474568	G/T	intronic	ARSB	G=0.988
5	139237820	rs114578583	G/A	intronic	NRG2	G=0.989
5	81291199	rs147143324	C/T	intronic	ATG10	C=0.959
5	78279820	rs163129	G/A	intronic	ARSB	G=0.867
5	81469580	rs16899380	A/T	intronic	ATG10	A=0.825
5	179677940	rs17683933	T/G	intronic	MAPK9	T=0.879
5	180670858	rs2241369	G/C	UTR5	GNB2L1	G=0.967
5	180663035	rs2261114	G/C	downstream	GNB2L1,TRIM41	G=0.622
5	78073245	rs3088247	C/A	UTR3	ARSB	C=0.749
5	78116136	rs339027	C/G	intronic	ARSB	C=0.802
5	78130051	rs339030	C/T	intronic	ARSB	C=0.783
5	115165913	rs35179959	C/A	UTR3	ATG12	C=0.813
5	78284877	rs4279321	A/G	intergenic	ARSB,DMGDH	A=0.882
5	78144104	rs55638484	T/C	intronic	ARSB	T=0.865
5	78126561	rs62377505	C/A	intronic	ARSB	C=0.762
5	179141391	rs62404346	A/C	intronic	CANX	A=0.588
5	139245762	rs6580288	A/G	intronic	NRG2	A=0.846
5	81438449	rs74793677	A/G	intronic	ATG10	A=0.987
5	139253738	rs77893406	G/A	intronic	NRG2	G=0.984
6	162141896	rs1018462	A/G	intronic	PARK2	A=0.658
6	117892414	rs11153675	G/C	intronic	GOPC	G=0.926
6	108880652	rs113490969	C/T	upstream	FOXO3	C=0.979
6	106694600	rs117077419	T/C	intronic	ATG5	T=0.978
6	162316655	rs117287596	T/C	intronic	PARK2	T=0.761

6	161794839	rs12194727	C/T	intronic	PARK2	C=0.799
6	41667037	rs13206766	C/T	intronic	TFEB	C=0.756
6	41656640	rs13213655	G/A	intronic	TFEB	G=0.912
6	106667994	rs2299864	C/T	downstream	ATG5	C=0.814
6	162319583	rs13437405	C/T	intronic	PARK2	C=0.975
6	162662827	rs138228179	G/T	intronic	PARK2	G=0.985
6	106745263	rs142985032	T/G	intronic	ATG5	T=0.988
6	161890191	rs143450375	T/C	intronic	PARK2	T=0.964
6	162859217	rs143750818	C/T	intronic	PARK2	C=0.971
6	162656566	rs147237284	G/A	intronic	PARK2	G=0.986
6	162011458	rs147775335	C/T	intronic	PARK2	C=0.984
6	36655513	rs148683340	C/T	downstream	CDKN1A	C=0.985
6	162249989	rs150958341	C/T	intronic	PARK2	C=0.973
6	162216058	rs187181677	A/G	intronic	PARK2	A=0.963
6	162442344	rs2851400	G/A	intronic	PARK2	T=0.648
6	44214488	rs34094794	C/T	intronic	HSP90AB1	C=0.897
6	44215784	rs34124063	A/T	intronic	HSP90AB1	T=0.683
6	162932218	rs34235347	C/A	intronic	PARK2	C=0.864
6	41702357	rs34313611	A/T	intergenic	PGC	A=0.907
6	162947874	rs34665989	G/A	intronic	PARK2	G=0.832
6	162447623	rs34706458	T/A	intronic	PARK2	T=0.930
6	44215556	rs34917612	G/A	intronic	HSP90AB1	G=0.953
6	162162658	rs35621098	G/A	intronic	PARK2	G=0.989
6	162896720	rs4709629	C/T	intronic	PARK2	T=0.530
6	161897689	rs61114332	C/T	intronic	PARK2	T=0.583
6	162752612	rs62430696	T/C	intronic	PARK2	T=0.986
6	161825925	rs62435929	T/A	intronic	PARK2	T=0.982
6	162265991	rs62436137	G/A	intronic	PARK2	G=0940
6	162117092	rs67965942	T/C	intronic	PARK2	T=0.890
6	162808350	rs6899614	C/G	intronic	PARK2	G=0.983

6	106728695	rs6906688	T/A	intronic	ATG5	T=0.907
6	162803587	rs6912641	C/A	intronic	PARK2	C=0.757
6	41666065	rs6916515	G/A	intronic	TFEB	G=0.858
6	162472401	rs73030487	T/C	intronic	PARK2	T=0.986
6	162292876	rs74950972	C/T	intronic	GOPC	C=0.982
6	162253773	rs75169534	T/G	intronic	PARK2	T=0.983
6	161801043	rs75786741	G/A	intronic	PARK2	G=0.972
6	162302879	rs76388920	T/C	intronic	PARK2	T=0.985
6	162234333	rs7739802	C/T	intronic	PARK2	T=0.535
6	161840889	rs7745681	C/T	intronic	PARK2	C=0.852
6	161864017	rs78224461	T/C	intronic	PARK2	C=0.893
6	162977857	rs79575917	C/A	intronic	PARK2	C=0.901
6	161952553	rs80011918	C/T	intronic	PARK2	T=0.975
6	162161666	rs1884158	T/C	intronic	PARK2	T=0.697
6	162609719	rs9295184	C/T	intronic	PARK2	G=0.598
6	161970752	rs9346864	A/G	intronic	PARK2	C=0.652
6	161847010	rs9355898	T/C	intronic	PARK2	C=0.704
6	162163506	rs9364611	G/A	intronic	PARK2	C=0.851
6	162278027	rs9364614	T/A	intronic	PARK2	T=0.503
6	161988513	rs9456684	C/T	intronic	PARK2	C=0.508
6	162606080	rs9458476	C/T	intronic	PARK2	C=0.949
6	41651447	rs9471625	A/T	intronic	PARK2	C=0.642
7	5230230	rs10273502	C/G	intronic	WIPI2	C=0.968
7	55180094	rs111744584	C/A	intronic	EGFR	C=0.984
7	6411978	rs113136758	G/A	intergenic	FAM220A,RAC1	G=0.943
7	55156673	rs117729456	G/A	intronic	EGFR	G=0.969
7	150723448	rs117897473	G/C	intergenic	ATG9B,ABCB8	G=0.961
7	55156803	rs202028278	A/G	intronic	EGFR	A=0.683
7	55213918	rs2072453	A/C	intronic	EGFR	C=0.548
7	55265354	rs2740763	A/T	intronic	EGFR	T=0.722

7	108215858	rs4727713	G/A	downstream	DNAJB9	G=0.917
7	55182730	rs4947977	G/A	intronic	EGFR	A=0.533
7	55200398	rs62459768	G/C	intronic	EGFR	G=0.937
7	151166463	rs73154847	A/G	intronic	RHEB	A=0.964
7	151205276	rs73154878	C/T	intronic	RHEB	C=0.970
7	55093086	rs78910000	T/C	intronic	EGFR	T=0.981
7	55082949	rs79015116	G/A	intergenic	LOC100996654,EGFR	G=0.984
7	55206861	rs887824	G/T	intronic	EGFR	G=0.921
7	105890056	rs9034	A/G	UTR3	NAMPT	C=0.673
7	105906643	rs929604	T/C	intronic	NAMPT	T=0.978
7	5260487	rs9987030	C/T	intronic	WIPI2	C=0.823
8	42143739	rs10099598	G/A	intronic	IKBKB	A=0.958
8	13044688	rs10108106	T/C	intronic	DLC1	T=0.574
8	13147046	rs116894784	C/T	intronic	DLC1	C=0.988
8	32178217	rs117070246	C/A	intronic	NRG1	C=0.905
8	31617362	rs117192588	T/C	intronic	NRG1	T=0.989
8	32473270	rs11780520	C/T	intronic	NRG1	C=0.877
8	31548094	rs12682592	G/A	intronic	NRG1	G=0.772
8	13080275	rs13250979	C/T	intronic	DLC1	C=0.913
8	32145300	rs139956466	C/T	intronic	NRG1	C=0.987
8	37883997	rs146163967	C/T	intergenic	ADRB3,EIF4EBP1	C=0.929
8	32571781	rs150171844	C/A	intronic	NRG1	C=0.971
8	12990734	rs17553593	C/A	UTR5	DLC1	C=0.731
8	32517515	rs17728839	A/G	intronic	NRG1	A=0.941
8	13272087	rs17817533	C/G	intronic	DLC1	C=0.920
8	32433685	rs2439299	A/C	intronic	NRG1	A=0.537
8	13270805	rs28642075	A/C	intronic	DLC1	C=0.540
8	31813471	rs327388	C/A	intronic	NRG1	C=0.979
8	32264790	rs4035324	A/T	intronic	NRG1	A=0.581
8	32474622	rs4535704	A/G	intronic	NRG1	A=0.634

8	128750540	rs4645959	A/G	exonic	MYC	A=0.953
8	32451286	rs4733363	A/T	intronic	NRG1	A=0.616
8	11699730	rs4841601	T/C	downstream	CTSB	T=0.659
8	13181803	rs62492886	C/T	intronic	DLC1	C=0.953
8	12990354	rs7013311	C/T	intronic	DLC1	C=0.969
8	13294495	rs74515132	C/T	intronic	DLC1	C=0.969
8	32175181	rs76825279	C/T	intronic	NRG1	C=0.891
8	32425752	rs77544541	C/A	intronic	NRG1	C=0.981
8	42131010	rs77896899	C/T	intronic	IKBKB	C=0.961
8	31537548	rs77929613	A/G	intronic	NRG1	A=0.968
8	13145046	rs78027969	G/A	intronic	DLC1	G=0.969
8	26253189	rs78067841	C/T	intronic	BNIP3L	C=0.681
8	32024592	rs7833041	T/G	intronic	NRG1	T=0.974
8	37892543	rs79396024	C/T	intronic	EIF4EBP1	C=0.979
8	32596669	rs79572378	G/A	intronic	NRG1	G=0.966
8	13275576	rs9657216	T/G	intronic	DLC1	G=0.632
9	90269214	rs10512188	T/C	intronic	DAPK1	T=0.882
9	90294733	rs11141943	C/G	intronic	DAPK1	C=0.981
9	90277099	rs117318552	C/G	intronic	DAPK1	C=0.968
9	135798121	rs117986467	G/A	intronic	TSC1	G=0.987
9	25676072	rs12555144	C/A	downstream	TUSC1	A=0.600
9	90108646	rs13296984	C/T	intergenic	C9orf170,DAPK1	G=0.527
9	90191415	rs147351813	T/C	intronic	DAPK1	T=0.986
9	90229542	rs17480679	G/A	intronic	DAPK1	G=0.959
9	21980744	rs3731227	G/A	intronic	CDKN2A	G=0.944
9	21991923	rs2811710	A/G	intronic	CDKN2A	A=0.619
9	25678639	rs61483294	G/A	UTR5	TUSC1	G=0.940
9	90262000	rs76327212	A/G	intronic	DAPK1	A=0.989
9	90120907	rs79213339	G/A	intronic	DAPK1	G=0.983
10	101287764	rs10883365	G/A	ncRNA_exonic	LINC01475	G=0.507

10	83697630	rs10883951	G/A	intronic	NRG3	G=0.769
10	83779609	rs10884044	G/C	intronic	NRG3	G=0.805
10	87431548	rs10887514	A/C	intronic	GRID1	A=0.735
10	88096047	rs10887578	G/C	intronic	GRID1	G=0.523
10	84672703	rs111525280	C/T	intronic	NRG3	C=0.940
10	83835232	rs11192402	C/A	intronic	NRG3	A=0.735
10	87887418	rs11201891	C/T	intronic	GRID1	C=0.970
10	83940514	rs114298958	C/T	intronic	NRG3	C=0.983
10	83839871	rs116895362	G/T	intronic	NRG3	G=0.987
10	87795058	rs117711043	C/T	intronic	GRID1	C=0.974
10	6519237	rs117775203	C/T	intronic	PRKCQ	C=0.969
10	87660149	rs12773496	T/C	intronic	GRID1	T=0.983
10	6494196	rs137882235	C/G	intronic	PRKCQ	C=0.876
10	83894117	rs140017377	A/G	intronic	NRG3	A=0.987
10	83677273	rs144352950	G/T	intronic	NRG3	G=0.967
10	71923311	rs144937416	G/A	intronic	SAR1A	G=0.987
10	90751882	rs148155729	T/A	ncRNA_exonic	FAS-AS1	T=0.967
10	83639836	rs148421664	T/C	intronic	NRG3	T=0.978
10	121416182	rs149103600	G/A	intronic	BAG3	G=0.976
10	84016070	rs17099664	T/C	intronic	NRG3	T=0.987
10	83875762	rs1739766	G/T	intronic	NRG3	T=0.536
10	83917680	rs1896508	A/G	intronic	NRG3	A=0.973
10	83747086	rs191339664	C/G	intronic	NRG3	C=0.989
10	87656075	rs1917143	T/A	intronic	GRID1	T=0.723
10	32336330	rs211289	T/G	intronic	KIF5B	T=0.966
10	133796291	rs2282056	T/C	upstream	BNIP3	T=0.814
10	89712473	rs2673832	G/A	intronic	PTEN	A=0.895
10	121407628	rs34135944	A/G	intergenic	TIAL1,BAG3	A=0.951
10	84570610	rs516285	C/G	intronic	NRG3	C=0.981
10	87938124	rs56064086	A/G	intronic	GRID1	A=0.937

10	87994091	rs61857817	C/T	intronic	GRID1	C=0.979
10	87865009	rs7082134	A/G	intronic	GRID1	G=0.646
10	87819622	rs71471187	T/C	intronic	GRID1	T=0.985
10	84649426	rs72821829	A/T	intronic	NRG3	A=0.954
10	89637201	rs74559740	T/A	intronic	PTEN	T=0.984
10	84053298	rs76164205	G/T	intronic	NRG3	G=0.967
10	84391145	rs77056587	A/G	intronic	NRG3	A=0.986
10	33252094	rs77677848	T/G	intergenic	ITGB1,NRP1	T=0.960
10	87680435	rs78333648	C/T	intronic	GRID1	C=0.881
10	87372193	rs7906330	C/T	intronic	GRID1	C=0.978
10	89716186	rs79909788	T/A	intronic	PTEN	T=0.973
10	87938800	rs876859	G/A	intronic	GRID1	G=0.958
10	90766596	rs9658750	A/G	intronic	FAS	A=0.855
11	72535083	rs112951181	A/C	intronic	ATG16L2	A=0.980
11	64965029	rs113295247	C/T	intronic	CAPN1	C=0.951
11	75560735	rs113472062	C/T	intronic	UVRAG	C=0.975
11	75533919	rs11605679	C/T	intronic	UVRAG	C=0.922
11	1788892	rs143309009	A/G	intergenic	CTSD,SYT8	A=0.989
11	64675148	rs143623527	G/A	intronic	ATG2A	G=0.984
11	1780704	rs149907533	A/G	intronic	CTSD	A=0.982
11	104908237	rs1699094	C/T	intergenic	CASP1,CARD16	C=0.957
11	104813100	rs34436514	A/G	downstream	CASP4	A=0.775
11	72537072	rs3765638	G/A	intronic	ATG16L2	G=0.810
11	64957016	rs4492832	C/G	intronic	CAPN1	G=0.813
11	1777969	rs61869049	C/T	intronic	CTSD	C=0.985
11	1773663	rs74362165	G/A	downstream	CTSD	G=0.918
11	46454428	rs77161239	G/A	intronic	AMBRA1	G=0.965
12	119623470	rs11064698	T/C	intronic	HSPB8	T=0.721
12	102273330	rs111753435	A/G	intronic	DRAM1	A=0.977
12	64884908	rs11175414	G/A	intronic	TBK1	G=0.984

12	119631086	rs117867844	G/A	intronic	HSPB8	G=0.986
12	121740564	rs144813769	G/A	intergenic	CAMKK2,ANAPC5	G=0.974
12	10371814	rs150976941	A/C	intronic	GABARAPL1	A=0.985
12	52462705	rs183332071	G/A	intergenic	NR4A1,ATG101	G=0.977
12	6643927	rs3741915	T/G	UTR5	GAPDH	T=0.742
12	102287753	rs4764664	G/T	intronic	DRAM1	G=0.566
12	10367376	rs58290036	C/T	intronic	GABARAPL1	C=0.931
12	6640443	rs71579330	C/T	intronic	NCAPD2	C=0.973
13	49036460	rs117540897	C/T	intronic	RB1	C=0.988
13	49023767	rs141276592	G/A	intronic	RB1	G=0.979
13	37608993	rs142078227	T/C	intronic	SUPT20H	T=0.981
13	113949751	rs9549380	G/A	intergenic	CUL4A,LAMP1	G=0.733
14	53165078	rs10151414	T/C	intergenic	ERO1A,PSMC6	C=0.534
14	94855831	rs1122629	G/A	intronic	SERPINA1	A=0.508
14	73472723	rs113290936	C/T	intronic	ZFYVE1	C=0.946
14	55844094	rs116917085	C/T	intronic	ATG14	C=0.963
14	73480161	rs138949716	C/T	intronic	ZFYVE1	C=0.984
14	67831839	rs148435097	T/G	intronic	EIF2S1	T=0.983
14	53119682	rs150650570	C/T	intronic	ERO1A	C=0.984
14	24663105	rs181951924	G/A	intronic	TM9SF1	G=0.856
14	55878617	rs185534171	G/A	upstream	ATG14	G=0.988
14	24665007	rs2234096	T/G	upstream	TM9SF1	T=0.897
14	53109716	rs35738167	T/G	UTR3	ERO1A	G=0.545
14	55834585	rs45470002	A/G	UTR3	ATG14	A=0.960
14	96746979	rs4900319	T/C	downstream	ATG2B	C=0.775
14	96814692	rs61985188	G/T	intronic	ATG2B	G=0.940
14	67827723	rs77603679	A/G	intronic	EIF2S1	A=0.976
14	67849857	rs78552927	C/A	intronic	EIF2S1	C=0.951
14	96797473	rs8012399	T/C	intronic	ATG2B	C=0.984
15	64284305	rs76685257	A/G	intronic	DAPK2	A=0.982

15	66157289	rs139615051	T/C	intergenic	DENND4A,RAB11A	T=0.979
16	28982517	rs12050928	T/C	intergenic	NFATC2IP,SPNS1	T=0.691
16	2123756	rs141280935	C/T	intronic	TSC2	C=0.983
16	2103686	rs144712953	T/C	intronic	TSC2	T=0.987
16	84754649	rs145998836	G/A	intronic	USP10	G=0.986
16	28505660	rs151234	G/C	upstream	APOBR	G=0.862
16	84771707	rs181455560	G/C	intronic	USP10	G=0.928
16	84808622	rs2303231	T/G	intronic	USP10	T=0.985
16	2259766	rs26858	A/G	intronic	BRICD5	A=0.506
16	2099232	rs3760042	A/G	intronic	TSC2	A=0.515
16	84744750	rs6564068	C/T	intronic	USP10	C=0.670
16	84745660	rs67729455	C/T	intronic	USP10	C=0.962
16	2103261	rs7185742	C/T	intronic	TSC2	C=0.989
16	84755825	rs7202154	A/C	intergenic	KLHL36,USP10	A=0.963
16	2130727	rs9923663	C/T	intronic	TSC2	C=0.671
17	73747520	rs113424540	G/A	intronic	ITGB4	G=0.963
17	78779170	rs113943264	C/A	ncRNA_exonic	LOC101928855	C=0.982
17	66418432	rs11652161	G/C	intronic	PRKAR1A,WIPI1	G=0.698
17	78865313	rs117713965	G/A	intronic	RPTOR	G=0.962
17	37860994	rs118036166	A/G	intronic	ERBB2	A=0.978
17	41319754	rs12451738	G/A	ncRNA_intronic	LOC101929767	G=0.980
17	48146368	rs12602101	T/C	intronic	ITGA3	T=0.900
17	80574532	rs12950772	A/G	intronic	WDR45B	A=0.730
17	80603191	rs12951801	T/A	intronic	WDR45B	T=0.965
17	78720001	rs138537551	C/T	intronic	RPTOR	C=0.934
17	62126615	rs140686727	C/T	intronic	ERN1	C=0.974
17	41356042	rs140827633	C/T	intronic	NBR1	C=0.987
17	79808522	rs144803881	G/C	intronic	P4HB	G=0.982
17	4588600	rs146174946	C/A	intronic	PELP1	C=0.982
17	78922107	rs149095681	G/A	intronic	RPTOR	G=0.977

17	76213642	rs17878683	A/G	intronic	BIRC5	A=0.967
17	78083155	rs187534497	G/A	intronic	GAA	G=0.983
17	48143686	rs188978084	T/C	intronic	ITGA3	T=0.985
17	73735326	rs2010043	C/A	intronic	ITGB4	C=0.880
17	41318153	rs2175956	G/C	ncRNA_intronic	LOC101929767	G=0.606
17	66428751	rs3897877	C/T	intronic	PRKAR1A,WIPI1	C=0.873
17	78524244	rs4627412	G/A	intronic	RPTOR	A=0.719
17	78938134	rs55677452	C/T	UTR3	RPTOR	C=0.967
17	79664583	rs55842573	C/T	intronic	HGS	C=0.942
17	74377161	rs56324625	G/C	intergenic	PRPSAP1,SPHK1	G=0.952
17	78883164	rs6565489	A/C	intronic	RPTOR	C=0.560
17	41332734	rs72831075	A/C	intronic	NBR1	A=0.903
17	78784728	rs72853735	A/G	intronic	RPTOR	A=0.865
17	78838310	rs74534309	G/T	intronic	RPTOR	G=0.759
17	78812175	rs746405	G/T	intronic	RPTOR	T=0.579
17	80585873	rs75359963	C/T	intronic	WDR45B	C=0.974
17	62156430	rs76959113	A/G	intronic	ERN1	A=0.967
17	7571752	rs78378222	T/G	UTR3	TP53	T=0.987
17	78878134	rs78927876	C/T	intronic	RPTOR	C=0.983
17	57864752	rs80178956	T/G	intronic	VMP1	T=0.974
17	78892647	rs34048269	G/A	intronic	RPTOR	G=0.788
17	48137168	rs9894097	C/T	intronic	ITGA3	C=0.900
18	60962957	rs113885940	T/G	intronic	BCL2	T=0.976
18	60805046	rs11872403	T/G	intronic	BCL2	T=0.543
18	60880068	rs189035660	T/C	intronic	BCL2	T=0.978
18	60988146	rs62098660	G/A	intergenic	BCL2,KDSR	G=0.524
18	60888801	rs72943089	G/A	intronic	BCL2	G=0.824
18	60931418	rs72945034	T/C	intronic	BCL2	T=0.902
18	60846430	rs74625348	G/C	intronic	BCL2	G=0.779
18	60977973	rs78803499	C/T	intronic	BCL2	C=0.961

19	36637605	rs17882574	A/G	intronic	CAPNS1	A=0.902
19	49378328	rs595474	C/T	intronic	PPP1R15A	C=0.889
19	3977762	rs74995777	G/A	ncRNA_intronic	MIR1268A	G=0.974
20	62163012	rs117890987	C/T	intronic	PTK6	C=0.963
20	33129984	rs143189192	G/A	intronic	PTK6	G=0.980
20	32399200	rs2626522	C/G	intergenic	DYNLRB1,MAP1LC3A	G=0.511
20	62159504	rs310644	G/A	upstream	TP53INP2	A=0.928
20	1364521	rs6041880	A/G	UTR5	CHMP4B	G=0.850
20	30286191	rs6060775	A/G	downstream	PTK6	A=0.561
20	62713640	rs6090042	G/A	ncRNA_intronic	FKBP1A-SDCBP2	A=0.911
20	33287782	rs6119510	T/A	intronic	BCL2L1	T=0.586
20	33129835	rs73103188	T/C	intronic	OPRL1	C=0.963
22	22132196	rs113229372	T/C	intronic	MAPK1	T=0.973
22	39919408	rs117826659	G/A	downstream	ATF4	G=0.980
22	22211605	rs11913063	T/C	intronic	MAPK1	T=0.949
22	22216376	rs140534946	G/A	intronic	MAPK1	G=0.982
22	36662718	rs140768119	A/G	UTR3	APOL1	A=0.917
22	51063070	rs141494339	T/C	UTR3	ARSA	T=0.973
22	22132403	rs28720995	G/A	intronic	MAPK1	G=0.568
22	41222684	rs55722734	G/A	intronic	ST13	G=0.989
22	22121276	rs61757965	G/A	intronic	MAPK1	G=0.982
22	18259071	rs8190251	C/T	upstream	LINC00528	C=0.964
22	18222567	rs8190330	G/A	intronic	BID	G=0.979

Abbreviations: SNP, single nucleotide polymorphism; Chr, Chromosome; MAF, minor allele frequency; UTR, untranslated region; CEU, Northern and Western Europeans
