# Supplementary material for: Polymorphisms within Autophagy-Related Genes as Susceptibility Biomarkers for Multiple Myeloma: A Meta-Analysis of Three Large Cohorts and Functional Characterization
Source: Int J Mol Sci. 2023 May 9;24(10):8500. doi: 10.3390/ijms24108500 (PMC10218542; doi:10.3390/ijms24108500)
Supplement: Supplementary file 1 [file ijms-24-08500-s001.zip › Sanchez-Maldonado_JM_(1). Supplementary_Table_4_List_of_serum-plasma-metabolites-analysed.pdf]

Supplementary Table 4. Serum and plasma metabolites measured in the HFGP cohort

Serum_markers

Panel	Assay	Uniprot ID	Units	OlinkID
Olink INFLAMMATION(v.3021)	4E-BP1	Q13541		OID00536
Olink INFLAMMATION(v.3021)	ADA	P00813		OID00560
Olink INFLAMMATION(v.3021)	ARTN	Q5T4W7		OID00526
Olink INFLAMMATION(v.3021)	AXIN1	O15169		OID00487
Olink INFLAMMATION(v.3021)	Beta-NGF	P01138		OID00519
Olink INFLAMMATION(v.3021)	CASP-8	Q14790		OID00550
Olink INFLAMMATION(v.3021)	CCL11	P51671		OID00505
Olink INFLAMMATION(v.3021)	CCL19	Q99731		OID00513
Olink INFLAMMATION(v.3021)	CCL20	P78556		OID00556
Olink INFLAMMATION(v.3021)	CCL23	P55773		OID00530
Olink INFLAMMATION(v.3021)	CCL25	O15444		OID00551
Olink INFLAMMATION(v.3021)	CCL28	Q9NRJ3		OID00539
Olink INFLAMMATION(v.3021)	CCL3	P10147		OID00532
Olink INFLAMMATION(v.3021)	CCL4	P13236		OID00498
Olink INFLAMMATION(v.3021)	CD244	Q9BZW8		OID00477
Olink INFLAMMATION(v.3021)	CD40	P25942		OID00542
Olink INFLAMMATION(v.3021)	CD5	P06127		OID00531
Olink INFLAMMATION(v.3021)	CD6	P30203		OID00499
Olink INFLAMMATION(v.3021)	CD8A	P01732		OID05124
Olink INFLAMMATION(v.3021)	CDCP1	Q9H5V8		OID00476
Olink INFLAMMATION(v.3021)	CSF-1	P09603		OID00562
Olink INFLAMMATION(v.3021)	CST5	P28325		OID00491
Olink INFLAMMATION(v.3021)	CX3CL1	P78423		OID00552
Olink INFLAMMATION(v.3021)	CXCL1	P09341		OID00496
Olink INFLAMMATION(v.3021)	CXCL10	P02778		OID00535
Olink INFLAMMATION(v.3021)	CXCL11	O14625		OID00486
Olink INFLAMMATION(v.3021)	CXCL5	P42830		OID00520
Olink INFLAMMATION(v.3021)	CXCL6	P80162		OID00534
Olink INFLAMMATION(v.3021)	CXCL9	Q07325		OID00490
Olink INFLAMMATION(v.3021)	DNER	Q8NFT8		OID01213
Olink INFLAMMATION(v.3021)	EN-RAGE	P80511		OID00541
Olink INFLAMMATION(v.3021)	FGF-19	O95750		OID00545
Olink INFLAMMATION(v.3021)	FGF-21	Q9NSA1		OID00512
Olink INFLAMMATION(v.3021)	FGF-23	Q9GZV9		OID00507
Olink INFLAMMATION(v.3021)	FGF-5	P12034		OID00509
Olink INFLAMMATION(v.3021)	Flt3L	P49771		OID00533
Olink INFLAMMATION(v.3021)	GDNF	P39905		OID00475
Olink INFLAMMATION(v.3021)	HGF	P14210		OID00522
Olink INFLAMMATION(v.3021)	IFN-gamma	P01579		OID05547
Olink INFLAMMATION(v.3021)	IL-1 alpha	P01583		OID00493
Olink INFLAMMATION(v.3021)	IL10	P22301		OID00528
Olink INFLAMMATION(v.3021)	IL-10RA	Q13651		OID00508
Olink INFLAMMATION(v.3021)	IL-10RB	Q08334		OID00515
Olink INFLAMMATION(v.3021)	IL-12B	P29460		OID00523
Olink INFLAMMATION(v.3021)	IL13	P35225		OID00525
Olink INFLAMMATION(v.3021)	IL-15RA	Q13261		OID00514
Olink INFLAMMATION(v.3021)	IL-17A	Q16552		OID00485
Olink INFLAMMATION(v.3021)	IL-17C	Q9P0M4		OID00483

Plasma_markers

Assay	Units
hsCRP	ug/mL
IL18bpx	pg/mL
Resistin	ng/mL
Leptin	ng/mL
Adiponectin	ug/mL
AAT	mg/mL
IL-1Ra_Q (Quantikine)	pg/mL
IL18 pg/mL	pg/mL
IL-1b pg/mL	pg/mL
IL-6 pg/mL	pg/mL
VEGF-A	pg/mL

Olink INFLAMMATION(v.3021)	IL18	Q14116	OID00501
Olink INFLAMMATION(v.3021)	IL-18R1	Q13478	OID00517
Olink INFLAMMATION(v.3021)	IL2	P60568	OID00495
Olink INFLAMMATION(v.3021)	IL-20	Q9NYY1	OID00537
Olink INFLAMMATION(v.3021)	IL-20RA	Q9UHF4	OID00489
Olink INFLAMMATION(v.3021)	IL-22 RA1	Q8N6P7	OID00516
Olink INFLAMMATION(v.3021)	IL-24	Q13007	OID00524
Olink INFLAMMATION(v.3021)	IL-2RB	P14784	OID00492
Olink INFLAMMATION(v.3021)	IL33	O95760	OID00543
Olink INFLAMMATION(v.3021)	IL4	P05112	OID00546
Olink INFLAMMATION(v.3021)	IL5	P05113	OID00559
Olink INFLAMMATION(v.3021)	IL6	P05231	OID00482
Olink INFLAMMATION(v.3021)	IL7	P13232	OID00478
Olink INFLAMMATION(v.3021)	IL8	P10145	OID00471
Olink INFLAMMATION(v.3021)	LAP TGF-beta-1	P01137	OID00480
Olink INFLAMMATION(v.3021)	LIF	P15018	OID00547
Olink INFLAMMATION(v.3021)	LIF-R	P42702	OID00511
Olink INFLAMMATION(v.3021)	MCP-1	P13500	OID00484
Olink INFLAMMATION(v.3021)	MCP-2	P80075	OID00549
Olink INFLAMMATION(v.3021)	MCP-3	P80098	OID00474
Olink INFLAMMATION(v.3021)	MCP-4	Q99616	OID00504
Olink INFLAMMATION(v.3021)	MMP-1	P03956	OID00510
Olink INFLAMMATION(v.3021)	MMP-10	P09238	OID00527
Olink INFLAMMATION(v.3021)	NRTN	Q99748	OID00548
Olink INFLAMMATION(v.3021)	NT-3	P20783	OID00554
Olink INFLAMMATION(v.3021)	OPG	O00300	OID00479
Olink INFLAMMATION(v.3021)	OSM	P13725	OID00494
Olink INFLAMMATION(v.3021)	PD-L1	Q9NZQ7	OID00518
Olink INFLAMMATION(v.3021)	SCF	P21583	OID00500
Olink INFLAMMATION(v.3021)	SIRT2	Q8IXJ6	OID00538
Olink INFLAMMATION(v.3021)	SLAMF1	Q13291	OID00502
Olink INFLAMMATION(v.3021)	ST1A1	P50225	OID00557
Olink INFLAMMATION(v.3021)	STAMBP	O95630	OID00558
Olink INFLAMMATION(v.3021)	TGF-alpha	P01135	OID00503
Olink INFLAMMATION(v.3021)	TNF	P01375	OID05548
Olink INFLAMMATION(v.3021)	TNFB	P01374	OID00561
Olink INFLAMMATION(v.3021)	TNFRSF9	Q07011	OID00553
Olink INFLAMMATION(v.3021)	TNFSF14	O43557	OID00506
Olink INFLAMMATION(v.3021)	TRAIL	P50591	OID00488
Olink INFLAMMATION(v.3021)	TRANCE	O14788	OID00521
Olink INFLAMMATION(v.3021)	TSLP	Q969D9	OID00497
Olink INFLAMMATION(v.3021)	TWEAK	O43508	OID00555
Olink INFLAMMATION(v.3021)	uPA	P00749	OID00481
Olink INFLAMMATION(v.3021)	VEGFA	P15692	OID00472
